# Sertraline exposure during development may impact post‐myocardial infarction survival in adult mice

**DOI:** 10.14814/phy2.70662

**Published:** 2025-11-14

**Authors:** Yongjun Lu, Elizabeth Kenkel, Kathy Zimmerman, Robert M. Weiss, Robert D. Roghair, Sarah E. Haskell

**Affiliations:** ^1^ Department of Pediatrics University of Iowa Carver College of Medicine Iowa City Iowa USA; ^2^ Bristol‐Myers Squibb Seattle Washington USA; ^3^ Department of Internal Medicine University of Iowa Carver College of Medicine Iowa City Iowa USA; ^4^ Department of Veteran Affairs Medical Center Iowa City Iowa USA

**Keywords:** cardiac function, developmental exposure, myocardial infarction, serotonin receptors, sertraline, sex‐specific effects

## Abstract

This study examines sex‐specific effects of developmental sertraline exposure on cardiac function and gene expression before and after myocardial infarction (MI) in mice. Female C57BL/6 mice (10 weeks) received intraperitoneal sertraline (5 mg/kg/day, *n* = 37) or saline (*n* = 20) before mating, during pregnancy, and postnatally to pups (1.5 mg/kg/day, postnatal Days 0–14). MI in offspring was induced at 10 weeks by left coronary artery ligation. Randomly chosen offspring (sham *n* = 8 and MI *n* = 26 per sex) underwent baseline echocardiography and at 10 weeks post‐MI if surviving. Serotonin‐ and estrogen‐related gene expression was analyzed. Before MI, sertraline‐exposed females had lower heart rate (649.1 ± 102.0 vs. 692.9 ± 38.4 bpm, *n* = 34), increased end‐systolic volume, and reduced ejection fraction (80.7 ± 6.3% vs. 83.9 ± 3.5%; *p* < 0.05). Exposed males also had lower heart rates (665.9 ± 32.7 vs. 683.3 ± 47.9 bpm, *n* = 34, *p* < 0.05). Post‐MI, both sexes remodeled similarly (scar size, ischemic‐zone fraction); sertraline‐exposed males had higher scar‐zone collagen (*p* < 0.05) and a nonsignificant lower survival trend than females. Sertraline altered serotonin‐related gene expression (*Htr2a*, *Htr2b*, *Slc6a4*), particularly in male sham mice. Developmental sertraline exposure induces sex‐specific cardiac changes, potentially affecting post‐MI outcomes, with males showing more structural and survival impairments.

## Graphical abstract

The impact of developmental sertraline exposure on post‐myocardial infarction (MI) outcomes.

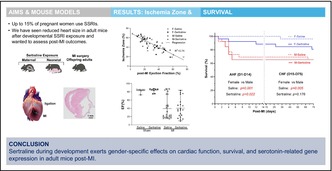

## 1 INTRODUCTION

Serotonin (5‐hydroxytryptamine, 5‐HT) plays a significant role in the cardiovascular system (Ayme‐Dietrich et al., 2017). Both animal and human data suggest that changes in 5‐HT concentration and signaling, that is, activation of the receptors, can lead to cardiovascular pathology (Ayme‐Dietrich et al., 2017; Côté et al., 2003; Mekontso‐Dessap et al., 2006; Nebigil et al., 2001, 2003; Qvigstad et al., 2005; Sauls et al., 2012). Many drugs targeting the serotonin transporter (SERT) and receptors are widely used in the general population, and may increase cardiovascular risks. Recent work has highlighted that SERT^−/−^ mice have pathological remodeling of the cardiac valves, myocardial fibrosis, and diminished ejection fraction (Castillero et al., 2023, 2025), which is more prominent in older adult mice (Castillero et al., 2025). The 5‐HT2B receptor is important in regulating cardiac hypertrophy and remodeling in left ventricular (LV) dysfunction and data have revealed a complex effect of the 5‐HT2B receptors in cardiomyocytes, cardiac fibroblasts and coronary vessels (Ayme‐Dietrich et al., 2017). Pharmacologic 5‐HT2B antagonism or tamoxifen‐inducible Cre‐mediated ablation of 5‐HT2B from cardiac fibroblasts improved remodeling post‐ myocardial infarction (Snider et al., 2021).

Selective serotonin reuptake inhibitors (SSRIs) are the most prescribed antidepressant (Latendresse et al., 2017). SSRIs prevent the reuptake of 5‐HT via SERT. We have previously demonstrated that chronic perinatal exposure to the antidepressant sertraline in mice resulted in reduced adult ventricular size and function, with decreased expression of the 5‐HT2B receptor (Haskell et al., 2017; Haskell, Hermann, et al., 2013a). We also observed modulation of 5‐HT signaling pathways through upregulated miRNAs, which corresponded with downregulation of *Htr2a*, *Htr2b*, and *Slc6a4* mRNAs and their proteins (Lu et al., 2024). While our whole pregnancy sertraline exposure model has largely demonstrated a subtle phenotype with decreased exercise performance as young adult mice (Haskell et al., 2017), we were curious how an additional later cardiovascular injury with myocardial infarction (MI) may impact the overall cardiovascular health of the offspring. In an earlier exposure model representing late trimester SSRI exposure, the offspring were provided protection against adult post‐MI dilation and reduction in cardiac function (Haskell, Peotta, et al., 2013b). However, the MI studies were performed in only males and the exposure model was more representative of maternal exposure rather than fetal exposure. Given our consistent findings of reduced ventricular size, 5‐HT_2B_ expression and cardiac function with perinatal exposure across animal models (Haskell et al., 2017; Kent et al., 2022; Lu et al., 2024), we sought to determine if this small heart phenotype from perinatal sertraline exposure would impact post‐MI outcomes utilizing a physiologically relevant whole pregnancy model and if sex would influence outcomes. We also assessed additional cardiac genes, including estrogen‐related, cellular function, and structural genes, because prior studies suggest these genes may modulate sex‐dependent cardiac remodeling and survival after MI. Including these genes allowed us to explore potential secondary pathways through which developmental sertraline exposure could influence post‐MI outcomes. With the use of SSRIs during pregnancy approaching 10%–15%, understanding the long‐term cardiovascular risks of this early‐life exposure is crucial. We hypothesized that sertraline exposure during development would impair post‐MI recovery in a sex‐dependent manner due to alterations in serotonin and estrogen signaling.

## 2 MATERIALS AND METHODS

### 2.1 Animals and sertraline exposure model

All experimental protocols and procedures fully met the standards set forth within the regulations of the Animal Welfare Act and the National Institutes of Health Guide for the Care and Use of Laboratory Animals and were approved by the Institutional Animal Care and Use Committee of the University of Iowa.

**FIGURE 1:**
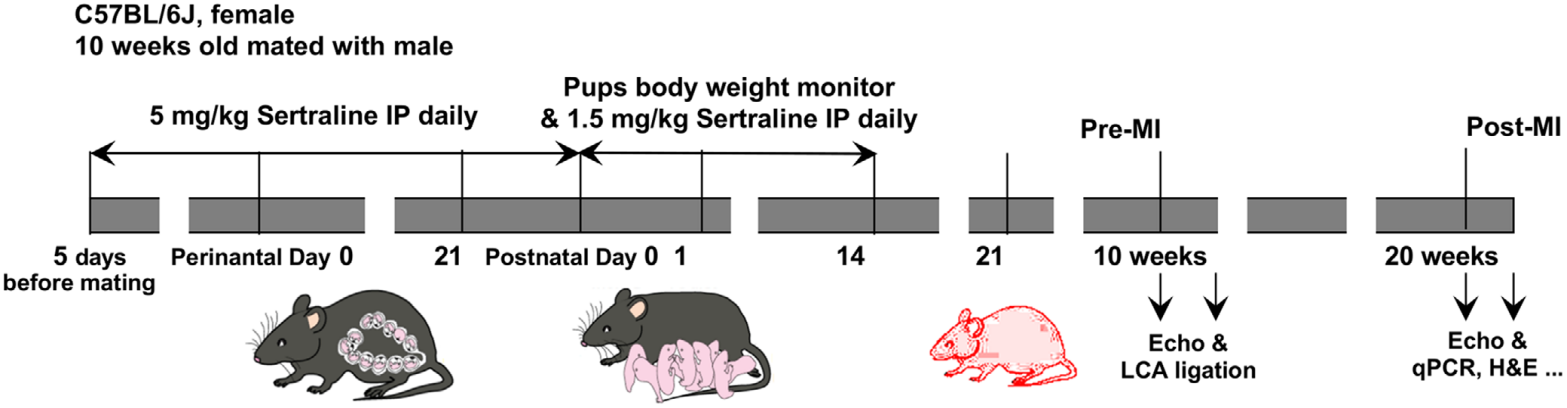
Schematic experimental mouse model of sertraline exposure & LCA ligation MI. Female C57BL/6J mice at the age of 10 weeks‐old were either received intraperitoneal (IP) saline or sertraline daily for 5 days prior mating, and daily throughout pregnancy to time of delivery. Pups were weighed immediately prior to daily administration of saline or sertraline from postnatal (PN) age 0–14 days. The mice were weaned at PN Day 21, and subjected to the first echocardiography at 10 weeks of age (pre‐MI). After the surgical LCA ligations, the mice were monitored daily until 20‐weeks‐old (post‐MI) when the second echocardiography was taken and the survived mice were euthanized for cardiac tissue analysis.

This study built on our previous investigation of maternal sertraline exposure and offspring cardiac function (Lu et al., 2024), in which adult C57BL/6J mice (10 weeks old) were obtained from Jackson Laboratory (Bar Harbor, ME). Female mice (*n* = 57: 37 sertraline and 20 saline) were mated to generate sertraline‐exposed offspring, following our established methods (Haskell et al., 2017; Meyer et al., 2018). Dams and their resulting pups were housed in a temperature‐controlled (22°C ± 2°C) facility with a strict 12‐h light/dark cycle and ad libitum access to food and water. Mice were maintained in complete autoclaved cage assemblies containing Cellu‐nest bedding (Shepherd Specialty Papers, Watertown, TN, USA) and fed standard rodent chow (NIH‐31 Irradiated Modified Open Formula Mouse/Rat Diet, Inotiv Inc., Indianapolis, IN, USA; Cat# 7913). As shown in Figure 1, female mice were administered intraperitoneal (IP) injections of either saline (0.9% Sodium Chloride Injection, USP, Hospira, Lake Forest, IL) at 10 mL/kg/day or sertraline (Cat# S6319, Sigma‐Aldrich, St. Louis, MO) at 5 mg/kg/day for 5 days prior to mating and throughout pregnancy until delivery.

After birth, the offspring received reduced doses of sertraline (1.5 mg/kg/day) or saline (3 mL/kg/day) from postnatal Day 0 to Day 14 (PN Days 0–14) to simulate placental drug transfer. This time frame corresponds to active cardiomyocyte proliferation in mice, similar to the third trimester in human gestation (Clubb & Bishop, 1984; Walsh et al., 2010). Mice were weaned on PN Day 21 and housed under standard care conditions with access to standard diets. Body weights were recorded on PN Days 0–14, PN Day 21, at 10 weeks of age, and at 20 weeks of age, continuing until euthanasia (10 weeks post‐MI surgery).

### 2.2 LCA ligation MI model

We performed a priori power analysis using the G*Power software before initiating experiments to determine the necessary sample size of mice to achieve a desired level of statistical power (Faul et al., 2007, 2009). For a *t*‐test comparing two independent groups, parameters included a significance level (α) of 0.05, power (1‐β) of 80%, and an allocation ratio of 1. A large effect size (Cohen's *d* = 0.8) required a minimum of 21 mice per group. Accounting for a 20% surgery‐induced mortality rate, group sizes were increased to 26. Control groups (non‐surgical) were set at *n* = 8, resulting in a total cohort of 152 mice divided into 12 subgroups based on sex and experimental conditions, consisting of equal numbers of male and female mice subjected to either sertraline exposure or saline: Non‐surgery (*n* = 4 per subgroup), Sham (*n* = 8 per subgroup), and MI (*n* = 26 per subgroup). The mice were randomly selected from the available cohort.

The permanent ligation of the left coronary artery (LCA) was used as a standard surgical method to induce MI in rodents (Gao et al., 2010; Johny & Dutta, 2022). This procedure involved blocking the left anterior descending artery, which supplies a significant portion of the left ventricle, resulting in infarction. All surgeries and analyses were performed in a blinded manner.

Ten‐week‐old male and female mice, exposed to either maternal and neonatal sertraline or saline, underwent LCA ligation. Sham‐operated mice at the same age underwent the same procedure without artery occlusion. The mice were anesthetized with 2.5% isoflurane (SKU: RXISO‐100, SHOPMEDVET, Mettawa, IL) and placed supine on a 37°C heating pad. After shaving and cleaning the surgical site, a midline or left thoracic incision was made to expose the thoracic cavity. The rib cage was carefully spread apart with a retractor to expose the heart. A 7–0 monofilament suture was tied tightly around the LCA, approximately 2 mm from its origin, to occlude blood flow. Successful occlusion was confirmed under a dissecting microscope by discoloration of the ischemic area.

The pericardium was closed with sutures, and the rib cage and skin were repositioned and sutured. Mice were monitored during recovery from anesthesia and given sustained‐release buprenorphine (2 mg/kg, Cat# IZ‐73000‐220,604, ZooPharm, Laramie, WY) for analgesia. The perioperative phase was defined as the first 5 h post‐surgery, during which mortality due to surgical complications was excluded from the study. Post‐operative care followed established protocols, including daily monitoring of body weight, food and water intake, and signs of distress or infection.

Surviving mice were euthanized, their hearts were weighed and processed for further analysis.

### 2.3 Echocardiography

Echocardiograms were performed using a 40 MHz linear array probe connected to a Vevo® 2100 imaging system (VisualSonics, Toronto, ON, Canada) as described in prior studies (Haskell et al., 2017; Lu et al., 2024). Mice were sedated with 0.1 mg midazolam (subcutaneous injection, Almaject®, Morristown, NJ). The anterior thorax, abdomen, and pelvis were shaved, and warmed ultrasound gel was applied to optimize the acoustic interface. Midazolam‐induced sedation is not expected to significantly lower heart rate or cause observable behavioral abnormalities aside from sedation (Berry et al., 2009).

Echocardiography was used to measure endocardial and epicardial areas during end‐diastole and end‐systole, respectively. Cardiac parameters, including ejection fraction (EF, %), mass (M, mg), stroke volume (SV, μL), and cardiac output (CO, μL/min), were calculated using the biplane area‐length method, adhering to the American Society for Echocardiography Guidelines. The ischemic zone fraction (IZfx%) was calculated from parasternal short‐axis cine loops, which provide a circumferential view of the LV suitable for global infarct size quantification. The endocardial length of the ischemic zone (akinetic or hypokinetic region) was traced at end‐systole and divided by the total LV endocardial circumference at end‐diastole, expressed as a percentage (Kang et al., 2006). Parasternal long‐axis views, in contrast, are more appropriate for assessing anterior or anteroseptal wall segments (Dann et al., 2022). LV mass was measured at the time of echocardiography and normalized to body weight for each mouse to account for size differences.

Offspring mice (sham *n* = 8 and MI *n* = 26 per sex) underwent baseline echocardiography at 10 weeks of age prior to MI. MI surgery was then performed at 10 weeks, and echocardiography was repeated 10 weeks post‐MI in surviving mice (survival numbers are reported in Table 1).

**TABLE 1:** Echocardiographic parameters of mouse groups.

		No surgery		Sham		MI	
		Saline	Sertraline	Saline	Sertraline	Saline	Sertraline
Female	*n*	33	33	7	8	25	21
	EDV (μL)	20.5 ± 3.8	22.3 ± 9.4	25.9 ± 4.9^!^	23.6 ± 2.8	100.8 ± 65.1^ǂ^	99.6 ± 77.0^ǂ^
	ESV (μL)	3.2 ± 0.6	4.5 ± 3.6*	4.3 ± 0.8	3.8 ± 0.7	73.8 ± 59.5^ǂ^	70.0 ± 67.1^ǂ^
	Mass (mg)	57.4 ± 8.0	55.5 ± 11.5	66.3 ± 10.0^!^	56.8 ± 5.5	82.4 ± 21.5	79.4 ± 26.9^ǂ^
	SV (μL)	17.2 ± 3.6	17.8 ± 6.8	21.6 ± 4.6^!^	19.8 ± 3.2	27.0 ± 9.4	29.7 ± 16.7
	CO (μL/min)	11.9 ± 2.5	11.0 ± 3.3	14.3 ± 2.9^!^	13.9 ± 2.1^!^	17.3 ± 6.5	18.6 ± 9.1
	EF (%)	83.9 ± 3.5	80.7 ± 6.3	83.0 ± 4.3^!^	83.3 ± 4.7	39.4 ± 25.8^ǂ^	40.5 ± 22.2^ǂ^
	FS (%)	16.5 ± 4.4	18.4 ± 5.1	19.9 ± 2.9	12.6 ± 2.7^!^	8.5 ± 4.7^ǂ^	7.6 ± 4.7^ǂ^
	HR (bmp)	692.9 ± 38.4	649.1 ± 102.0	697.7 ± 40.4	698.5 ± 24.4	647.8 ± 63.6^ǂ^	628.0 ± 49.9^ǂ^
	IZfx (%)	–	–	–	–	48.5 ± 2.9 (*n* = 20)	43.4 ± 3.2 (*n* = 17)
Male	*n*	33	33	8	8	17	17
	EDV (μL)	27.0 ± 6.8^§^	26.6 ± 10.3^§^	35.9 ± 9.9^! §^	45.8 ± 20.7^! §^	109.8 ± 84.5^ǂ^	104.5 ± 56.1^ǂ^
	ESV (μL)	4.1 ± 0.9	4.4 ± 3.0^§^	7.2 ± 3.6^!^	9.9 ± 4.8^!^	77.6 ± 85.9^ǂ^	71.9 ± 53.6^ǂ^
	Mass (mg)	66.7 ± 8.8^§^	67.1 ± 11.2^§^	80.3 ± 12.9^! §^	85.4 ± 23.7^! §^	92.5 ± 23.3	89.7 ± 17.5
	SV (μL)	22.9 ± 6.5^§^	22.20 ± 8.0^§^	28.7 ± 6.9^!^	35.8 ± 16.2^!§^	32.3 ± 10.3	32.6 ± 9.6
	CO (μL/min)	15.6 ± 4.3^§^	14.8 ± 5.3^§^	17.2 ± 2.3^!^	19.5 ± 8.7	20.8 ± 6.7	21.0 ± 7.2
	EF (%)	84.2 ± 3.8^§^	83.7 ± 3.7^§^	80.8 ± 5.4^!^	78.6 ± 2.4^! §^	47.8 ± 29.8^ǂ^	39.9 ± 22.4^ǂ^
	FS (%)	16.5 ± 4.4	17.4 ± 6.7	15.7 ± 3.3^§^	14.3 ± 3.3	8.9 ± 5.3^ǂ^	8.6 ± 5.0^ǂ^
	HR (bmp)	683.3 ± 47.9	665.9 ± 32.7*	643.5 ± 55.0	577.0 ± 82.5^!^	637.1 ± 77.6	623.9 ± 105.5
	IZfx (%)	–	–	–	–	51.6 ± 4.5 (*n* = 11)	47.9 ± 3.1 (*n* = 14)

*Note*: Four symbols were used to indicate statistically significant differences between two groups: (!) for *Sham* versus *No surgery*, (ǂ) for *MI* versus. *Sham*, (§) for *Female* versus *Male*, and (*) for *Sertraline* versus *Saline*. The numbers of mice for IZFx measurements were lower because some mice did not develop myocardial infarction (no detectable IZFx post‐surgery).

Abbreviations: CO, cardiac output; EDV, end‐diastolic volume; EF, ejection fraction; ESV, LV end‐systolic volume; FS, fractional shortening; HR, heart rate; IZfx, ischemic zone fraction; LV, Left ventricular; Mass, LV mass; SV, stroke volume.

### 2.4 Histochemical stains

Longitudinal histological sections from 16 mice were analyzed (*n* = 4 with no surgery, *n* = 4 with sham surgery, *n* = 8 with MI surgery). For hematoxylin and eosin (H&E) staining, hearts were perfused with PBS, fixed in 10% neutral‐buffered formalin, embedded in paraffin, and sectioned at 4–6 μm. Longitudinal sections along the apex–base axis were prepared to capture the full extent of infarcted myocardium.

For Masson's trichrome staining, three longitudinal sections per heart were obtained at 300 μm intervals starting from the mid‐ventricle. Sections were processed using a standard protocol, in which nuclei were stained with Weigert's hematoxylin, cytoplasm and muscle fibers with Biebrich scarlet–acid fuchsin, and collagen fibers with aniline blue. Stained sections were imaged using bright‐field microscopy (Olympus BX‐61, Tokyo, Japan) using CellSens software.

To validate echocardiographic scar quantification, representative male mice from the saline and sertraline groups were selected based on IZfx% values of ~57%–60% at 10 weeks post–LCA ligation. At sacrifice, hearts from these mice were sectioned at comparable mid‐ventricular levels and stained with H&E and Masson's trichrome. Infarct size was quantified by the endocardial circumference method: the infarcted arc length was traced in ImageJ and expressed as a percentage of the total LV endocardial perimeter. Scar‐zone collagen content was further assessed from trichrome‐stained sections by ImageJ color deconvolution, expressed as collagen‐positive area relative to total LV myocardial area.

Previous studies have demonstrated that echocardiographic assessment of myocardial infarction size in mice closely correlates with histological measurements (Dann et al., 2022). Histopathology, regarded as the reliable gold standard for evaluating infarct area, was therefore used to validate ultrasound‐based quantification (Takagawa et al., 2007). In this study, echocardiography served as the primary tool for assessing functional deficits in infarcted myocardium, with histology of representative sections providing complementary validation of structural scar, thereby strengthening confidence in the evaluation of myocardial remodeling.

### 2.5 qPCR for mRNA expression in mouse hearts

Quantitative polymerase chain reaction (qPCR) was used to evaluate mRNA expression in cardiac tissues, following established protocols as previously described (Lu et al., 2024). Hearts were randomly selected from each experimental group, and LV tissue was collected from the same anatomical region in all mice to ensure consistency. For qPCR analysis, *n* = 4 mice per group were used for most experimental groups; in the no‐surgery group, *n* = 3. In post‐MI hearts, samples were taken from non‐infarcted LV regions, with scar tissue excluded from RNA extraction. The LV samples were homogenized, and RNA was extracted using TRIzol™ Reagent (Cat# 15596018, Invitrogen, Carlsbad, CA). RNA samples were treated with DNase I (Cat# AM2222, Invitrogen) to remove residual DNA contamination. The NanoDrop™ ND‐2000 spectrophotometer (ThermoFisher Scientific, Wilmington, DE) was used to check the quality and quantity of the final RNA samples measured at 230, 260, and 280 nm. RNAs were reversely transcribed into cDNA using the AffinityScript™ QPCR cDNA Synthesis Kit (Cat# 600559, Agilent Technologies, Santa Clara, CA) according to the manufacturer's protocol.

qPCR was performed on a CFX96 Real‐Time System (Bio‐Rad, Hercules, CA) using primers (sequences in Table S1, purchased from IDT, Coralville, IA) and Brilliant SYBR Green QPCR Master Mix (Cat# 600828, Agilent Technologies). SYBR Green I, a dsDNA binding dye, quantified amplicon levels by monitoring fluorescence emission throughout the PCR process. Gene expression was normalized to *Gapdh* and analyzed using the ΔΔCT method.

*Gapdh*, commonly used for sample normalization, has faced criticism as a housekeeping gene in myocardial studies (Brattelid et al., 2010; Ruiz‐Villalba et al., 2017; You et al., 2013). Using the geNorm Excel program, reference genes like *H2afz*, *Myh6*, *Ppia*, and *Gapdh* were evaluated. Results from these genes closely matched those of *Gapdh* alone (Lu et al., 2024; Vandesompele et al., 2002). Thus, *Gapdh* was selected for normalization after careful analysis. Primer efficiencies ranged from 95%–110% (Table S1), supporting the ∆∆Ct method's validity. mRNA profiles remained consistent whether analyzed with a single *Gapdh* reference (∆∆Ct) or multiple genes (*Pfaffl* method) (Pfaffl, 2001).

### 2.6 Statistical analysis

Continuous variables were presented as mean ± SD. Statistical analyses included unpaired two‐tailed *t*‐tests and log‐rank (Mantel‐Cox) tests for survival analysis. For each measured data set, normality was assessed using the Shapiro–Wilk test (*p* > 0.05 indicating normal distribution). Based on the results, an unpaired *t*‐test was used when both groups were normally distributed, while the Mann Whitney *U* test was applied if one or both groups were non‐normally distributed. Statistical significance was defined as *p* < 0.05, *p* < 0.01, or *p* < 0.001. All computations were performed using Prism 10 (GraphPad, Boston, MA).

## 3 RESULTS

### 3.1 Induction of MI via LCA ligation

**FIGURE 2:**
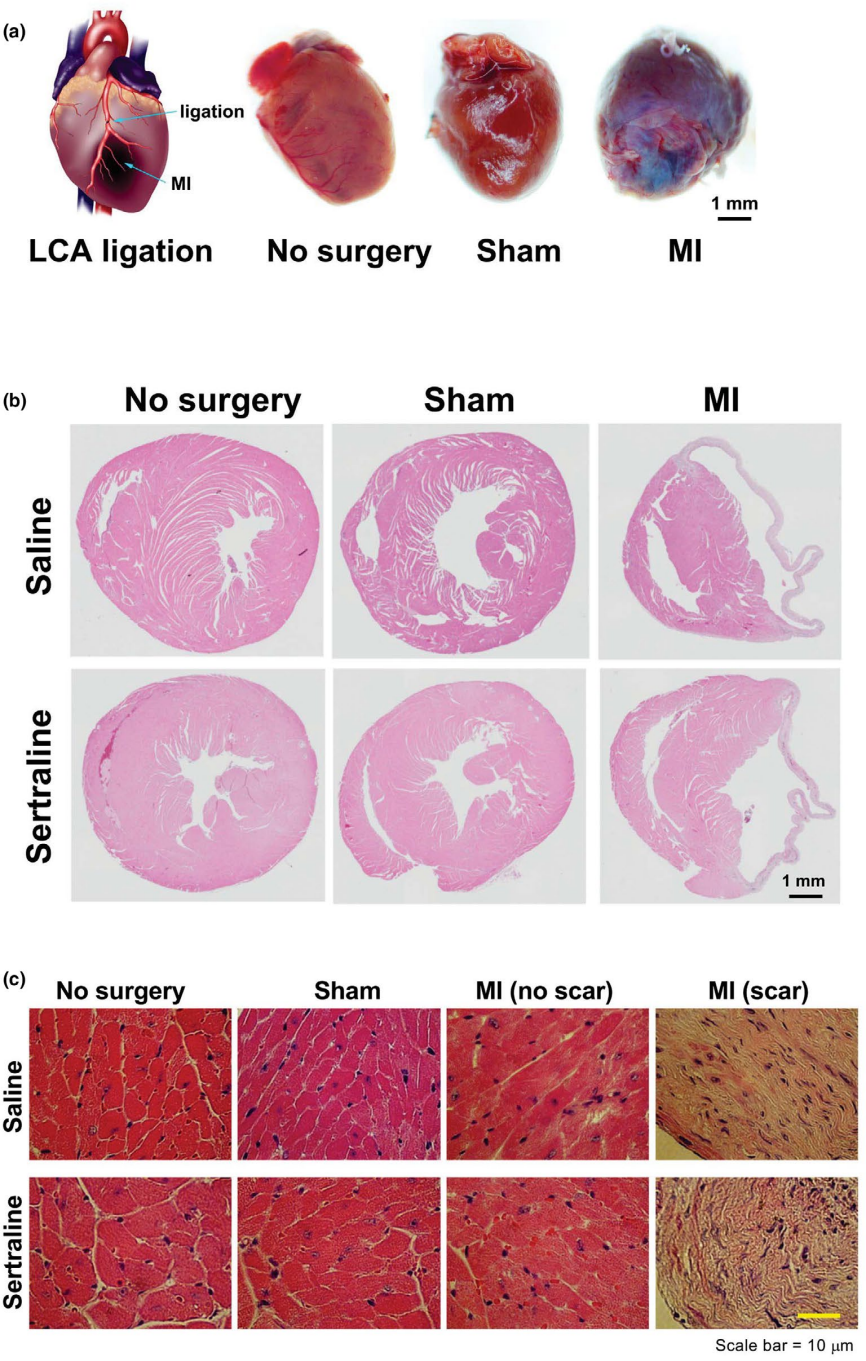
Representative images of hearts from male mice subjected to left coronary artery (LCA) ligation‐induced myocardial infarction (MI) with either sertraline exposure or saline. Ten weeks post‐surgery, hearts were excised, and tissue sections were stained with hematoxylin and eosin (H&E). Panel (a) depicts a schematic of mouse LCA ligation alongside images of mouse hearts without surgery (*n* = 4), with sham surgery (*n* = 4), and with MI surgery (*n* = 8). Panel (b) showcases H&E‐stained sections of mouse hearts, while Panel (c) provides a zoomed‐in view of the heart sections, highlighting areas without scars and those with scars in MI samples. Scale bars are included for reference.

Representative heart images for each subgroup were depicted in Figure 2. Cardiac morphology differences across groups were confirmed via H&E staining. Mice subjected to MI surgery exhibited infarct injuries and fibrotic scarring localized to the infarction zones (Figure 2b,c, right panels).

Echocardiographic analysis showed no overall differences in LV mass between sertraline‐ and saline‐exposed mice (Table S2), indicating that developmental sertraline exposure did not alter baseline cardiac morphology.

**FIGURE 3:**
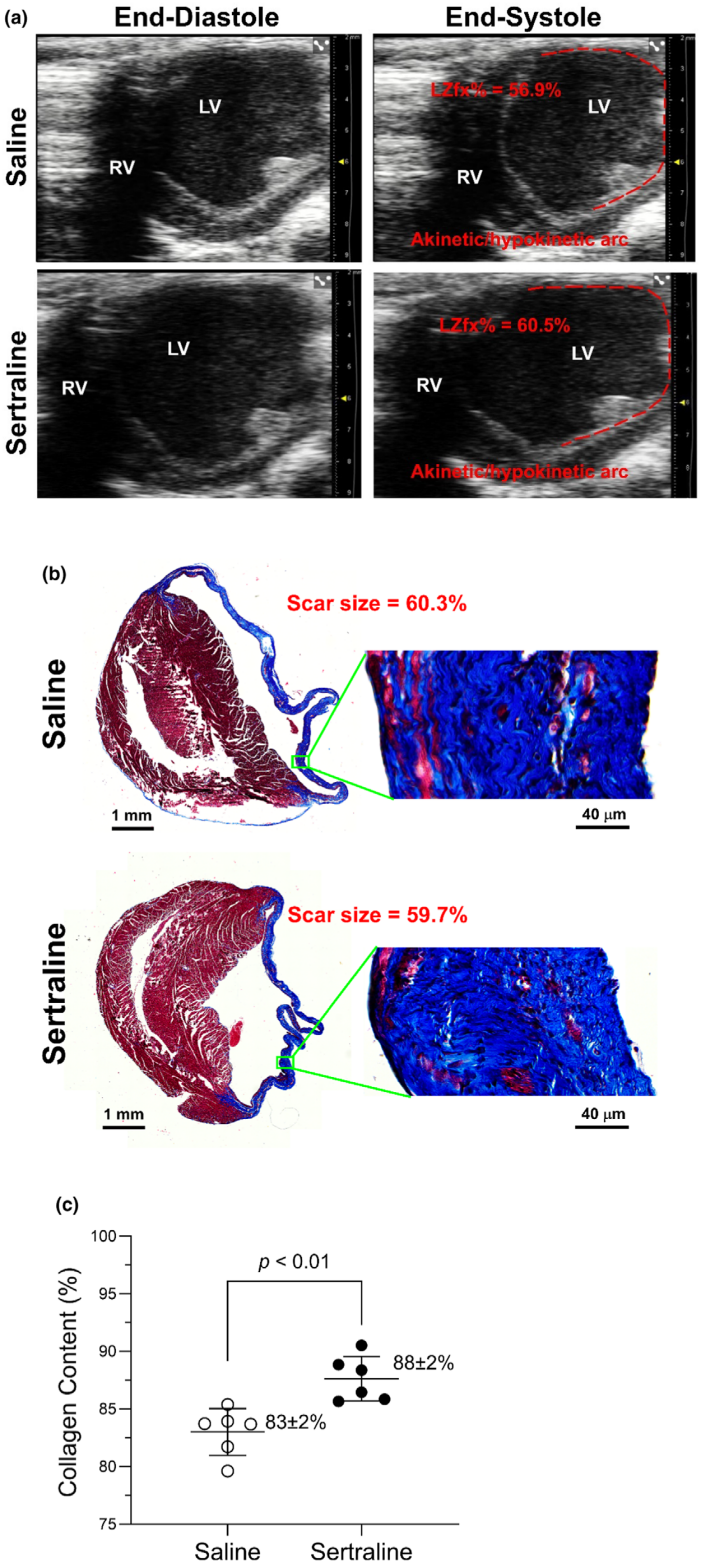
Echocardiographic IZfx% correlates with histological scar assessment in representative mice. (a) Parasternal short‐axis echocardiographic images at diastole (left) and systole (right). IZfx% was determined from cine loops by tracing akinetic segments, with still frames shown here for illustration. Dashed red lines delineated the ischemic zones. (b) Corresponding longitudinal Masson's trichrome–stained heart sections showing infarct size. Whole‐LV sections are displayed at low magnification with inset zooms highlighting scar morphology. (c) Collagen content quantified from longitudinal mid‐ventricular sections spaced 300 μm apart (6 section per mouse). Representative images are shown for saline‐ and sertraline‐treated mice. Together, these data illustrate the concordance between functional IZfx% (from echocardiography movies) and structural scar evaluation, as well as the effect of sertraline on post‐MI collagen deposition.

Masson's trichrome‐stained sections and corresponding quantification for representative mice are shown in Figure 3. Echocardiographic IZfx% served as the primary functional measure of infarct size. Longitudinal histological sections from 8 MI mice spanning the IZfx% range were analyzed (0%, *n* = 2; 25%, *n* = 1; 50%–60%, *n* = 5; data not shown). Across these mice, IZfx% closely reflected scar size: animals with IZfx% = 0 had no detectable scar, whereas higher IZfx% values were associated with progressively larger scars. In the representative examples, IZfx% (saline 56.9% vs. sertraline 60.5%) closely matched histological scar fractions (60.3% vs. 59.7%, Δ < 1%), supporting the reliability of IZfx% as the primary outcome. Group comparisons revealed no significant differences in IZfx% between saline‐ and sertraline‐treated mice. In contrast, collagen content analysis demonstrated significantly higher scar‐zone collagen in sertraline‐treated mice compared with saline controls (*p* < 0.01), indicating that sertraline selectively exacerbates post‐MI collagen deposition without altering overall infarct size.

### 3.2 Sex‐dependent survival curves

The cause of death in our study was inferred based on post‐MI timing, observed clinical signs, and gross pathological findings at necropsy. Mice that died within the first week post‐MI frequently exhibited signs consistent with acute heart failure, including labored breathing, lethargy, and cyanosis, with necropsy often revealing pleural effusion and pulmonary congestion. Deaths occurring beyond 4 weeks post‐MI were attributed to chronic heart failure when progressive weight loss, reduced activity, ascites, and ventricular dilation were observed. Although these findings provide insight into likely causes of mortality, other factors such as arrhythmias, thromboembolism, or scar rupture may have contributed but were not systematically assessed.

**FIGURE 4:**
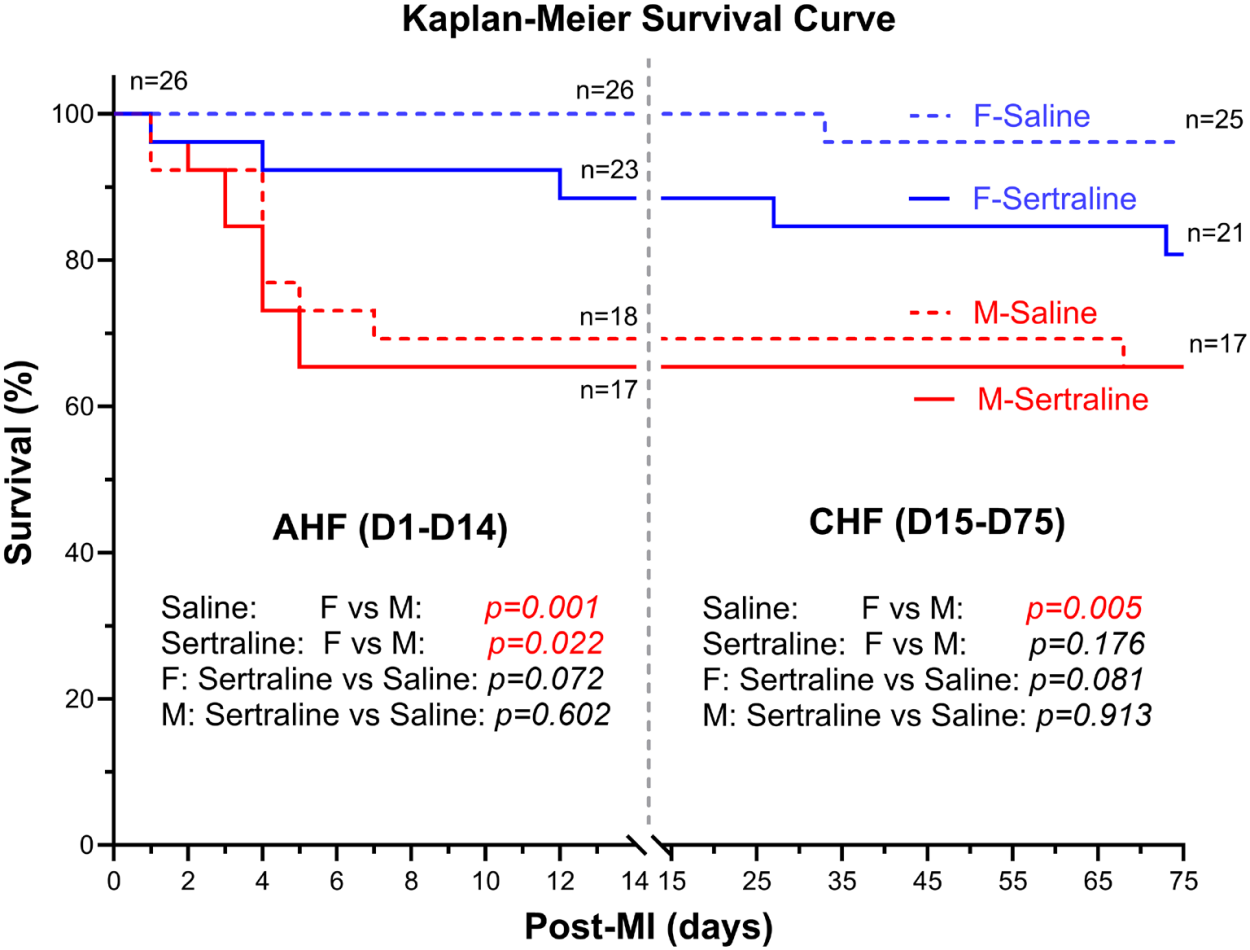
The survival curve of mice following left coronary artery (LCA) ligation reveals that early‐life sertraline exposure leads to a sex‐dependent risk of myocardial infarction (MI) in offspring mice during both the acute heart failure (AHF, D1–D14) and chronic heart failure (CHF, D15–D75) stages. *p*‐values for comparisons were included.

The survival rates of mice following LCA ligation were monitored daily, and the resulting survival curves are depicted in Figure 4. Early‐life exposure to sertraline resulted in a sex‐dependent risk of MI in offspring mice during the acute heart failure (AHF, D1–D14) stage. Sertraline‐treated females had better survival during the AHF stage compared to sertraline‐treated males (*p* < 0.05). Female mice in saline‐treated groups had better survival rates during both the AHF stage (*p* < 0.01) and the chronic heart failure (CHF, D15–75) stage (*p* < 0.05) than male saline‐treated mice. Sertraline‐treated females had trends toward lower survival during the AHF and CHF stages compared to saline‐treated females (AHF *p* = 0.072, CHF *p* = 0.081). No survival differences were observed in male mice between sertraline and saline groups during either stage (*p* > 0.05).

### 3.3 Ischemic zone and MI incidence

The ischemic zone fraction (IZFx%) was used to evaluate MI, with IZFx% >30% indicative of MI. As shown in Figure S1, MI successfully occurred in 77% of the saline‐treated male and female groups, 85% of sertraline‐treated females, and 92% of sertraline‐treated males. An inverse correlation between IZFx% and EF was observed (*R* ^2^ = 0.74), unaffected by sex or treatment.

To assess whether infarct size affected the analyzed outcomes, post‐MI mice were stratified into small‐ and large‐infarct groups across multiple cutoff points (<30%, <40%, <50%, and >30%, >40%, >50%, >60%). No significant differences between sertraline‐ and saline‐treated animals were observed in any subgroup, and the main conclusions remained unchanged (Table S2).

### 3.4 MI's impact on LV remodeling and cardiac function

LV remodeling was evident 10 weeks post‐MI, as reflected by significant enlargement of LV end‐diastolic volume (EDV) and LV end‐systolic volume (ESV) in both male and female mice compared to no surgery or sham groups regardless of saline or sertraline exposure (Table 1). These changes indicate an intense remodeling process and irreversible myocardial tissue loss following MI. EF and fractional shortening (FS), two key indicators of cardiac function and efficiency, were significantly reduced following myocardial infarction due to ischemic cardiomyopathy and ventricular dilation.

LV mass analysis showed sex‐ and treatment‐dependent differences. In females, normalized LV mass was significantly higher in MI compared with sham mice under both saline and sertraline conditions (*p* < 0.05), whereas no significant differences were observed in males (Table S3). Before normalization, only the female‐sertraline group showed significantly greater LV mass in MI compared with sham (Table 1). Normalization also eliminated sex‐based differences observed in sham groups, where males displayed higher LV mass than females regardless of treatment. Importantly, no significant differences were detected between sertraline‐ and saline‐treated animals within the same surgical group.

Sertraline exposure led to a significant FS reduction in the female sham surgery group compared to the no surgery group. Perinatal sertraline exposure led to reduced HR in non‐surgery females and males. These subtle sertraline‐related effects do not impact post‐MI outcomes positively or negatively. Individual measurements and means for all groups are shown in Figure S2.

### 3.5 Serotonin‐related gene expression

**FIGURE 5:**
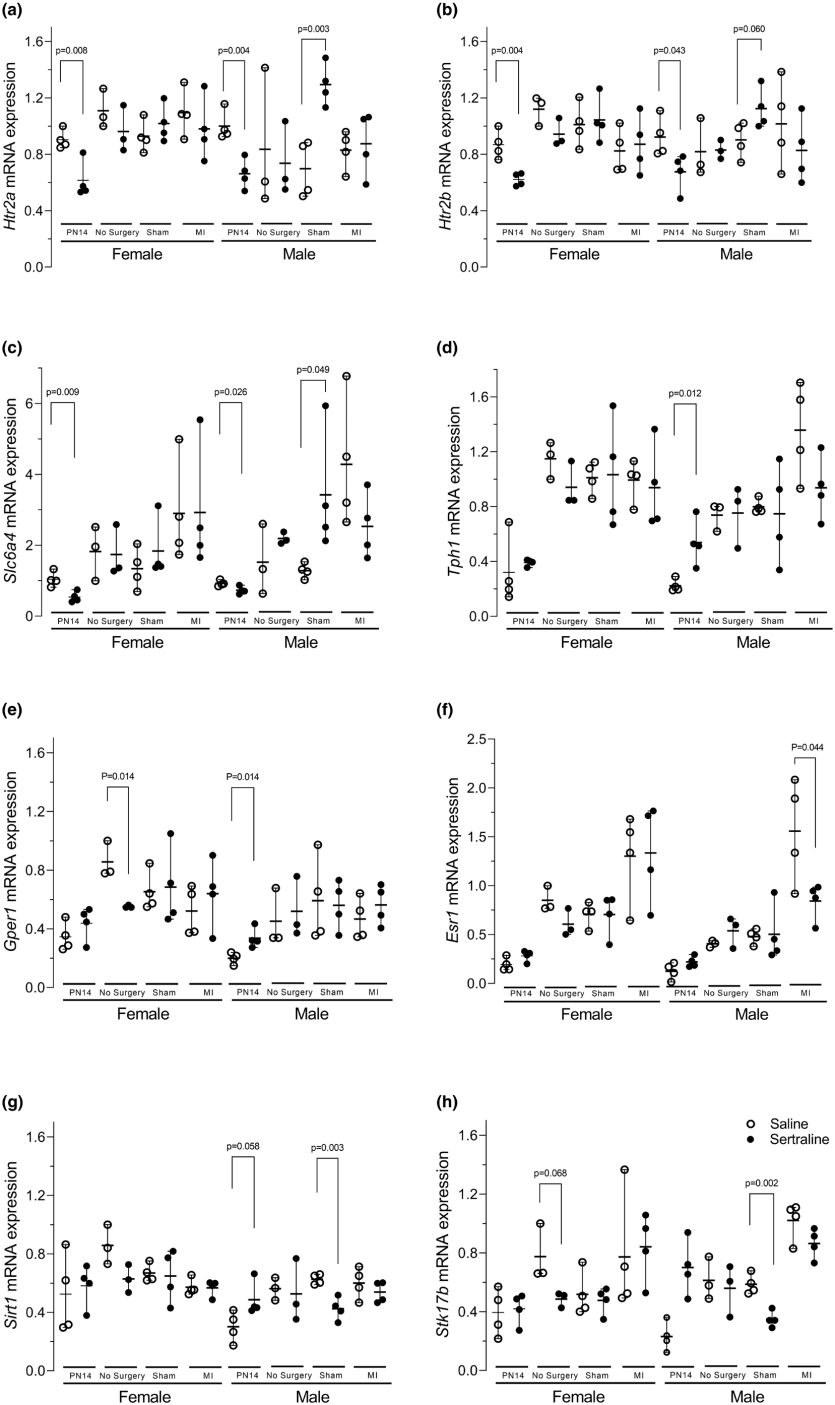
mRNA expression of 5‐HT signaling, estrogen signaling and other genes in cardiac tissues of female and male mice at different stages including on PN day 14 (*n* = 4), no surgery (*n* = 3), sham (*n* = 4) and MI surgery (*n* = 4). Expression levels are shown for (a) *Htr2a*, (b) *Htr2b*, (c) *Slc6a4*, (d) *Tph1*, (e) *Ger1*, (f) *Esr1*, (g) *Sirt1*, and (h) *Stk17b*. The effects of sertraline versus saline in each group were analyzed using an unpaired two‐tailed *t*‐test, with *p*‐values indicated in the graphs.

Serotonin‐related gene expression was measured in cardiac tissues on postnatal Day 14 (PN Day 14) and in adulthood at 20 weeks following no surgery, sham surgery or MI. As shown in Figure 5, sertraline exposure led to significant downregulation of *Htr2a, Htr2b*, and *Slc6a4* at baseline on PN 14. Sham surgery male groups showed significant upregulation of *Htr2a, Htr2b*, and *Slc6a4*. This trend was also observed in female sham surgery groups though not significant. Tryptophan hydroxylase 1 (*Tph1*) was upregulated in sertraline‐treated males on PN14, with no changes observed in females. Those baseline changes suggest that developmental SSRI exposure can alter cardiac transcriptional profiles independent of ischemic injury. These findings are exploratory and may indicate an underlying molecular phenotype that could influence cardiac responses to subsequent injury.

### 3.6 Sex‐specific gene expression

Sex‐based differences were noted in gene expression as shown in Figure 5. *Gper1* was significantly downregulated in sertraline‐treated non‐surgical females but upregulated in PN day 14 males. Estrogen receptor 1 (*Esr1*) increased in male MI groups, with this effect counteracted by sertraline. *Sirt1* expression increased in sertraline‐treated PN day 14 males but decreased in sham surgery males. Apoptosis‐related *Stk17b* (*DRAK2*) was downregulated in sertraline‐treated no surgery females and sham surgery male groups.

### 3.7 Sertraline effects on other cardiac genes

Sertraline exposure altered additional cardiac genes in male tissues involved in various cellular processes, including metabolism (Walsh et al., 2013), cell proliferation and differentiation (Cargnello & Roux, 2011), regulation of immune responses and inflammation (Parameswaran & Patial, 2010), and various aspects of cardiac physiology (Figure S3) (Abeyrathna & Su, 2015; Averill et al., 2012; Euler, 2015; Fontes et al., 2015; MacLennan & Kranias, 2003; Ozdemir et al., 2017; Sagiv & Portman, 2021). *Cyp1a1* was upregulated in sertraline males compared to saline males, and *Erk1* was upregulated in male saline compared to female saline (Figure S3H,I respectively), while *Tnfα* was reduced in sertraline males compared to saline males (Figure S3J). No significant changes were observed in seven other genes (*Akt1*, *Cd24a*, *Gnb3*, *IL6*, *Pln*, *S100a9*, *Tgfβ1*) with the results illustrated in Figure S3A–G.

## 4 DISCUSSION

The present study explores the effects of early‐life exposure to sertraline and subsequent adult cardiovascular stress caused by MI on cardiac structure, function, and gene expression within a murine model. This research builds upon prior studies that investigated the influence of early‐life sertraline or saline exposure on adult cardiovascular disease by inducing MI in these mice. However, this study takes a more clinically relevant approach by employing sertraline doses that better mimic fetal exposure (<10 ng/mL) (Meyer et al., 2018) rather than the higher levels (18.9 ng/mL) used in our earlier models (Haskell, Hermann, et al., 2013a; Haskell, Peotta, et al., 2013b). Moreover, the inclusion of both male and female mice enables the identification of sex‐specific effects, a departure from our previous work (Haskell, Hermann, et al., 2013a; Haskell, Peotta, et al., 2013b). These distinctions make this study uniquely positioned to provide deeper insights into the interplay between early‐life sertraline exposure and adult cardiovascular disease, with specific attention to sex‐dependent outcomes.

The findings reveal profound alterations in cardiac structure and function due to MI, as evidenced by significant increases in LV EDV, LV ESV, and a nonsignificant trend toward higher LV mass. These structural changes indicate extensive LV remodeling, a hallmark of the pathological sequelae following MI (Braunwald, 2013). Such remodeling is consistent with ischemic cardiomyopathy, characterized by ventricular dilation and impaired contractility. Additionally, the observed reduction in EF underscores diminished pumping efficiency as a result of infarct‐induced myocardial tissue loss (Mann & Bristow, 2005). Despite these significant changes, early‐life sertraline exposure appeared to have no substantial impact on the extent of LV remodeling or function post‐MI. These results diverge from findings in our previous study, where postnatal sertraline exposure led to decreased shortening fraction and increased LV volumes in both sertraline‐ and saline‐exposed mice. Interestingly, saline‐exposed mice exhibited greater LV dilatation and worsening cardiac function post‐MI, despite comparable infarct sizes across groups. This discrepancy may stem from differences in sertraline exposure levels as the earlier study utilized a model that reflected maternal rather than fetal sertraline exposure.

One of the novel aspects of this study was the inclusion of both sexes, allowing for a detailed investigation into sex‐specific outcomes. A striking observation was the significant sex‐dependent survival disparity following MI. Female mice demonstrated higher survival rates than their male counterparts, aligning with human studies that report lower heart failure risk in premenopausal women compared to men (Magnussen et al., 2019; Ziaeian et al., 2017). This survival advantage in female mice may be attributed to protective mechanisms involving estrogen signaling pathways and inflammation regulation. It has been reported that the synthesis of specialized pro‐resolving mediators plays a key role in post‐MI inflammation, with male mice exhibiting increased biosynthesis of these mediators, whereas female mice show elevated levels of epoxyeicosatrienoic acids (Pullen et al., 2020). These sex‐specific inflammatory responses suggest the presence of protective factors in females, possibly mediated by estrogen signaling. Haider et al. reported that males exhibit higher myocardial sympathetic tone and more pronounced adverse left ventricular remodeling following cardiac injury compared to females (Haider et al., 2025). Rizkallah et al. quantified sex‐specific differences in cardiac remodeling and function using cardiac magnetic resonance, showing prognostic implications of these disparities in cardiomyopathy (Rizkallah et al., 2025). Collectively, these findings suggest that females may maintain better post‐MI outcomes due to more efficient repair processes, lower sympathetic stress, and attenuated adverse remodeling.

Additional studies have indicated that males are more prone to post‐MI arrhythmias, mitral valve dysfunction, and scar rupture, which could contribute to higher mortality, while females benefit from protective mechanisms that preserve cardiac structure and function. For instance, a study by van Veen et al. discussed how sex‐specific differences in cardiac structural remodeling and therapy influence outcomes in heart failure (Kessler et al., 2019). Furthermore, another study by van Veen et al. highlighted how sex‐specific differences in cardiac structural remodeling and therapy influence outcomes in heart failure (Aimo et al., 2021). These findings underscore the need for sex‐specific approaches in the treatment and management of MI.

Interestingly, the survival advantage of female mice over male mice present in the acute phase for both saline and sertraline‐treated female mice disappeared in sertraline‐treated females in the chronic heart failure phase. This finding highlights sex‐specific protective mechanisms despite the potential adverse effects of sertraline exposure, further emphasizing the need for additional research into these complex interactions.

At the molecular level, the serotonin signaling pathway emerged as a significant target of interest. Key serotonin receptor genes: *Htr2a*, *Htr2b*, and *Slc6a4*, exhibited sex‐dependent modulation by sertraline exposure. Notably, mRNA expression of these genes was significantly reduced in both male and female mice exposed to sertraline compared to saline on PN 14. Interestingly, within the sham group of male mice, sertraline exposure led to an upregulation of these genes, while no discernible differences were observed post‐MI. Recent studies have highlighted the role of serotonin receptor subtypes in cardiac pathophysiology. For instance, cardiomyocyte 5‐HT_2A_ receptor activation has been implicated in myocardial ischemia–reperfusion injury through mechanisms involving mitochondrial reactive oxygen species production and platelet‐activating factor receptor signaling (Jin et al., 2023). Similarly, 5‐HT_2B_ receptor expression in cardiac fibroblasts has been linked to scar formation, adverse remodeling, and impaired cardiac function following MI (Snider et al., 2021). In this study, decreased *Htr2b* mRNA expression in sertraline‐exposed mice may explain the absence of differences in scar formation between saline‐ and sertraline‐exposed groups. Additionally, reduced *Htr2a* and *Htr2b* expression at baseline in sertraline‐exposed mice may contribute to the blunting of estrogen's protective effects, particularly in female mice. Indeed, previous research has suggested that decreased 5‐HT_2B_ receptor levels can impair estrogen‐mediated cardioprotection during hypoxia/reoxygenation (Dhaibar et al., 2021). These findings, combined with the better survival rates observed in females compared to males post‐MI, support the hypothesis that serotonin signaling pathways and their interaction with estrogen signaling may play a crucial role in mediating sex‐specific cardiovascular outcomes.

Although serotonin‐related gene expression differences were primarily observed in sham‐operated animals, these baseline transcriptional alterations may influence the heart's response to ischemic injury. Developmental sertraline exposure appears to induce an underlying molecular phenotype that could modify susceptibility to post‐MI remodeling and survival, even if subtle changes were not statistically detectable in post‐MI groups due to limited sample size. These results should be interpreted cautiously but provide a rationale for future mechanistic studies exploring how early‐life SSRI exposure shapes cardiac resilience.

Estrogen's protective effects against cardiovascular disease (CVD) have been well‐documented (Iorga et al., 2017; Wake & Yoshiyama, 2009), particularly in premenopausal women, where it is believed to confer reduced CVD risk through mechanisms involving estrogen receptor signaling (Menazza & Murphy, 2016; Ueda et al., 2020). Activation of estrogen receptor alpha (Esr1) has been shown to mitigate infarct size, reduce cardiomyocyte apoptosis, suppress inflammation and oxidative stress, enhance vasodilation, and promote neovascularization (Wake & Yoshiyama, 2009). This study revealed sex‐specific alterations in the expression of *Gper1*, *Esr1*, *Sirt1*, and *Stk17b*, suggesting intricate crosstalk between neurotransmitter and hormone pathways that warrants further investigation. The only significant finding post‐MI was *Esr1* mRNA expression in male mice and all male mice had worse outcomes post‐MI regardless of treatment. The significance of these findings needs further investigation with larger numbers to help understand the molecular underpinnings of cardiac remodeling, function, and MI outcomes.

Several limitations in our study should be acknowledged. First, the findings presented are limited to surviving mice following the MI model involving LCA ligation surgery. The use of the permanent LCA ligation model in this study offers valuable insights but is associated with relatively high surgery‐related mortality rates. The potential impact of sertraline exposure on the initial development and progression of MI remains unclear. Compared to our prior results, mice were given versed for sedation in this study whereas our previous echocardiograms were performed under isoflurane. Versed enhances the dominance of sympathetic activity in the cardiac autonomic nervous system during conscious sedation (Win et al., 2005). It is possible both methodological differences contributed to the higher heart rates seen in this study compared to our previous studies. As such, it will be difficult to make a direct comparison between the echocardiography results reported here and those in our prior studies. Our current study is limited in that the cause of death is inferred by post‐MI timing, observed clinical signs, and gross pathological findings at necroscopy. Furthermore, we did not elucidate the underlying mechanisms, including the roles of arrhythmias, mitral prolapse, and scar rupture, as possible contributions to the observed sex differences in survival. Finally, while the study explored serotonin and estrogen signaling pathways in relation to MI, it represents preliminary work that requires further investigation to elucidate the full spectrum of interactions between these pathways and their implications for sex‐specific cardiovascular disease.

## 5 CONCLUSION

This study provides significant insights into the complex interplay between MI, sertraline exposure, and sex, particularly regarding their effects on cardiac structure, function, and gene expression in a murine model. Sex‐specific survival disparities were observed, suggesting potential protective mechanisms in female mice, while gene expression analysis revealed sex‐dependent responses, especially in serotonin signaling and estrogen‐related genes. These findings underscore the importance of considering sex‐specific and stage‐specific effects of sertraline in MI progression and recovery. Importantly, the results highlight the need to carefully evaluate post‐MI acute survival.

## FUNDING INFORMATION

This research was financially supported by the National Institutes of Health (grants K08HL141528, S10OD019941, S10OD0038119), the Children's Miracle Network, and the Stead Family Department of Pediatrics.

## CONFLICT OF INTEREST

The authors declare no conflicts of interest.

## Supporting information


**Table S1.** qPCR primers for mouse genes.
**Table S2.** Subgroup analyses for all echocardiography parameters by infarct size: sertraline versus saline comparisons.
**Table S3.** Analysis of LV mass by echocardiography, normalized to body weight.
**Figure S1.** (a) The MI incidences were determined from echocardiograms with the criteria of >30% for MI incidence. (b) Ischemic zone fraction (IZFx) is proportional to post‐MI EF with correlation coefficient *R* ^2^ = 0.74. No significant differences were observed between sertraline and saline groups, and between female and male groups (*p* > 0.05). Mouse numbers used in each group were given in the figure.
**Figure S2.** MI significantly increased LV remodeling in mouse hearts when compared to mouse hearts of no surgery group whereas no significant effects of sertraline (Se) versus saline (Sa) were observed. (a) LV end‐diastolic volume (EDV), (b) LV end‐systolic volume (ESV), and (c) LV mass (Mass). The functional parameters were calculated including (d) Stroke Volume (SV), (e) Cardiac Output (CO), (f) Ejection Fraction (EF) and (g) Fractional Shortening (FS). (h) Heart Rate (HR). All measured data points were shown, with the mouse number indicated in the figure. Statistical analyses were reported in the main text and Table 1.
**Figure S3.** mRNA expression other genes in cardiac tissues of female and male mice post‐MI (*n* = 4), which play critical roles in various aspects of cardiac physiology. (a) *Akt1*, (b) *Cd24a*, (c) *Gnb3*, (d) *IL6*, (E) *Pln*, (f) *S100a9*, (g) *Tgfβ1*, (h) *Cyp1a1*, (i) *Ekr1*, and (j) *Tnfα*. The effects of sertraline versus saline in each group were analyzed by unpaired & two‐tails *t*‐test, and the *p*‐values were given in graphs.

## Data Availability

The data that supports the findings of this study is available in the Supporting Information S1 of this article, and is available from the corresponding author upon reasonable request.
