# Supplementary material for: Sertraline exposure during development may impact post‐myocardial infarction survival in adult mice
Source: Physiol Rep. 2025 Nov 14;13(22):e70662. doi: 10.14814/phy2.70662 (PMC12618208; doi:10.14814/phy2.70662)
Supplement: Supplementary file 1 — Table S1. qPCR primers for mouse genes. Table S2. Subgroup analyses for all echocardiography parameters by infarct size: sertraline versus saline comparisons. Table S3. Analysis of LV mass by echocardiography, normalized to body weight. Figure S1. (a) The MI incidences were determined from echocardiograms with the criteria of >30% for MI incidence. (b) Ischemic zone fraction (IZFx) is proportional to post‐MI EF with correlation coefficient R 2 = 0.74. No significant differences were observed between sertraline and saline groups, and between female and male groups (p > 0.05). Mouse numbers used in each group were given in the figure. Figure S2. MI significantly increased LV remodeling in mouse hearts when compared to mouse hearts of no surgery group whereas no significant effects of sertraline (Se) versus saline (Sa) were observed. (a) LV end‐diastolic volume (EDV), (b) LV end‐systolic volume (ESV), and (c) LV mass (Mass). The functional parameters were calculated including (d) Stroke Volume (SV), (e) Cardiac Output (CO), (f) Ejection Fraction (EF) and (g) Fractional Shortening (FS). (h) Heart Rate (HR). All measured data points were shown, with the mouse number indicated in the figure. Statistical analyses were reported in the main text and Table 1. Figure S3. mRNA expression other genes in cardiac tissues of female and male mice post‐MI (n = 4), which play critical roles in various aspects of cardiac physiology. (a) Akt1, (b) Cd24a, (c) Gnb3, (d) IL6, (E) Pln, (f) S100a9, (g) Tgfβ1, (h) Cyp1a1, (i) Ekr1, and (j) Tnfα. The effects of sertraline versus saline in each group were analyzed by unpaired & two‐tails t‐test, and the p‐values were given in graphs. [file PHY2-13-e70662-s001.docx]

| No. | Gene | GenBank Access | Primer’s sequence | Efficiency (%) | R^2^ |
| --- | --- | --- | --- | --- | --- |
| 1 | *Akt1*  (136 bp) | NM_009652.4 | Forward: GGACTACTTGCACTCCGAGAAG  Reverse: CATAGTGGCACCGTCCTTGATC | 103.1 | 0.9923 |
| 2 | *Cd24a*  (111 bp) | NM_009846.2 | Forward: ACATCTGTTGCACCGTTTCCCG  Reverse: CAGGAGACCAGCTGTGGACTG | 98.4 | 0.9839 |
| 3 | *Cyp1a1*  (147 bp) | NM_001136059.2 | Forward: CATCACAGACAGCCTCATTGAGC  Reverse: CTCCACGAGATAGCAGTTGTGAC | 97.5 | 0.9818 |
| 4 | *Erk1*  (129 bp) | NM_011952.2 | Forward: GGCTTTCTGACGGAGTATGTGG  Reverse: GTTGGAGAGCATCTCAGCCAGA | 104.3 | 0.9945 |
| 5 | *Esr1*  (153 bp) | NM_007956.5 | Forward: TCTGCCAAGGAGACTCGCTACT  Reverse: GTGCATTGGTTTGTAGCTGGAC | 107.2 | 0.9980 |
| 6 | *Gapdh*  (153 bp) | NM_001289726.1 | Forward: CATCACTGCCACCCAGAAGACTG  Reverse: ATGCCAGTGAGCTTCCCGTTCAG | 104.6 | 0.9997 |
| 7 | *Gnb3*  (128 bp) | NM_013530.1 | Forward: TGTTGCCGCTTCCTGGATGACA  Reverse: AGGCTCATGCAGTCACCAGTGT | 101.4 | 0.9825 |
| 8 | *Gper1*  (113 bp) | NM_007810.4 | Forward: CCACATAGTCAACCTTGCAGC  Reverse: CGTCTTCTGCTCCACATAGAGC | 105.7 | 0.9996 |
| 9 | *H2afz*  (131 bp) | NM_016750.3 | Forward: GGCCGTATTCATCGACACCTGA  Reverse: GACGCATTTCCTGCCAACTCAAG | 109.9 | 0.9976 |
| 10 | *Htr2a*  (127 bp) | NM_172812.3 | Forward: CTGATGTCACTTGCCATAGCTG  Reverse: AGGTAAATCCAGACGGCACAG | 100.9 | 0.9960 |
| 11 | *Htr2b*  (145 bp) | NM_008311.3 | Forward: GTGATGCCGATTGCCCTCTTG  Reverse: ATAGCGGTCCAGGGAAATGGCA | 96.2 | 0.9978 |
| 12 | *Il6*  (116 bp) | NM_031168.2 | Forward: TACCACTTCACAAGTCGGAGGC  Reverse: CTGCAAGTGCATCATCGTTGTTC | 98.6 | 0.9956 |
| 13 | *Myh6*  (126 bp) | NM_001164171.1 | Forward: GCTGGAAGATGAGTGCTCAGAG  Reverse: CCAGCCATCTCCTCTGTTAGGT | 106.4 | 0.9986 |
| 14 | *Pln*  (117 bp) | NM_001141927.1 | Forward: GGACCAAAGGAACTTGCCAGCT  Reverse: CAACAGGCAGCCAAATGTGAGC | 105.37 | 0.9905 |
| 15 | *S100a9*  (128 bp) | NM_009114.3 | Forward: TGGTGGAAGCACAGTTGGCAAC  Reverse: CAGCATCATACACTCCTCAAAGC | 96.8 | 0.9944 |
| 16 | *Sirt1*  (133 bp) | NM_019812.3 | Forward: GGAGCAGATTAGTAAGCGGCTTG  Reverse: GTTACTGCCACAGGAACTAGAGG | 105.3 | 0.9990 |
| 17 | *Slc6a4*  (141 bp) | NM_010484.2 | Forward: GTTGATGCTGCGGCTCAGATCT  Reverse: GAAGCTCGTCATGCAGTTCACC | 99.9 | 0.9935 |
| 18 | *Stk17b*  (150 bp) | NM_133810.3 | Forward: AGACCAACAGCAGAATCCTGCC  Reverse: ACAGGAGGACTTGGAGGTCTTC | 106.9 | 0.9983 |
| 19 | *Ppia*  (112 bp) | NM_008907.2 | Forward: CATACAGGTCCTGGCATCTTGTC  Reverse: AGACCACATGCTTGCCATCCAG | 107.7 | 0.9998 |
| 20 | *Tgfβ1*  (107 bp) | NM_011577.2 | Forward: TGATACGCCTGAGTGGCTGTCT  Reverse: CACAAGAGCAGTGAGCGCTGAA | 107.4 | 0.9941 |
| 21 | *Tnfα*  (139 bp) | NM_013693 | Forward: GGTGCCTATGTCTCAGCCTCTT  Reverse: CCATAGAACTGATGAGAGGGAG | 101.8 | 0.9976 |
| 22 | *Tph1*  (138 bp) | NM_009414.3 | Forward: TGTTGACTGCGACATCAGCCGA  Reverse: GAAACCAAGGGACAGTCTCCA | 100.4 | 0.9775 |

Supplementary Table 1. qPCR primers for mouse genes

Supplementary Table 2. Subgroup analyses for all echocardiography parameters by infarct size:

sertraline vs. saline comparisons.

| Cutoff | Mouse numbers for comparison | | | | Statistical result |
| --- | --- | --- | --- | --- | --- |
|  | Female vs Male | | Sertraline vs Saline | |  |
|  | Sertraline | Saline | Female | Male |  |
| All points | 17 vs 14 | 20 vs 11 | 17 vs 20 | 14 vs 11 | All comparison NS |
| <30% | 3 vs 1 | 2 vs 1 | 3 vs 2 | 1 vs 1 | NA |
| <40% | 9 vs 4 | 6 vs 2 | 9 vs 6 | 4 vs 2 | All comparison NS |
| <50% | 11 vs 7 | 8 vs 5 | 11 vs 8 | 7 vs 5 | All comparison NS |
| >30% | 14 vs 13 | 18 vs 10 | 14 vs 18 | 13 vs 10 | All comparison NS |
| >40% | 9 vs 10 | 14 vs 9 | 9 vs 14 | 10 vs 9 | All comparison NS |
| >50% | 6 vs 7 | 12 vs 6 | 6 vs 12 | 7 vs 6 | All comparison NS |
| >60% | 2 vs 3 | 4 vs 4 | 2 vs 4 | 3 vs 4 | All comparison NS |

Supplementary Table 3. Analysis of LV mass by echocardiography, normalized to body weight.

|  |  | Sham | | MI | |
| --- | --- | --- | --- | --- | --- |
|  |  | Saline | Sertraline | Saline | Sertraline |
| Female | n | 7 | 8 | 25 | 21 |
|  | Mass (mg) | 66.29±10.05 | 56.83±5.49 | 82.40±21.46 | 79.37±26.94^ǂ^ |
|  | Body Weight (g) | 26.54±1.30 | 22.16±0.88 | 25.49±3.03 | 23.71±2.48 |
|  | Normalized Mass  (Mass/BW, mg/g) | 2.49±0.34 | 2.56±0.20 | 3.29±0.98^ǂ^ | 3.34±1.07^ǂ^ |
| Male | n | 8 | 8 | 17 | 17 |
|  | Mass (mg) | 80.33±12.93^§^ | 85.38±23.65^§^ | 82.46±23.32 | 89.66±17.51 |
|  | Body Weight (g) | 33.94±2.69 | 30.40±2.52 | 31.31±1.44 | 30.60±2.24 |
|  | Normalized Mass  (Mass/BW, mg/g) | 2.38±0.42 | 2.81±0.75 | 2.96±0.79 | 2.94±0.60 |

**Note 1: Echocardiographic analysis showed no significant differences in LV mass, either absolute or normalized to body weight, between sertraline- and saline-exposed mice, indicating that sertraline did not alter gross cardiac morphology.**

**Note 2: Two symbols** were used to indicate statistically significant differences between two groups: **(§)** for Female vs. Male, and (ǂ) for *MI vs. Sham*.

Supplementary Figure 1.

A.


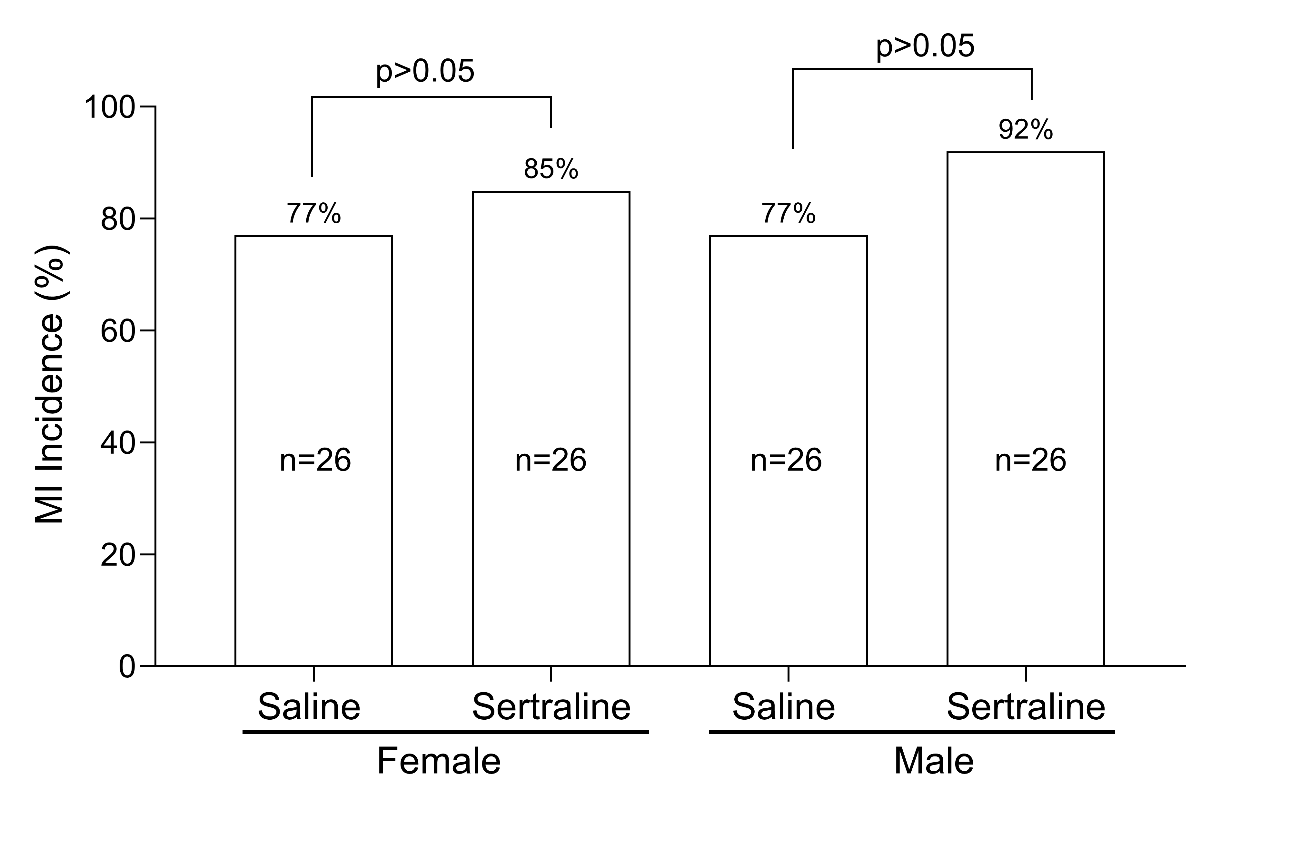


B.


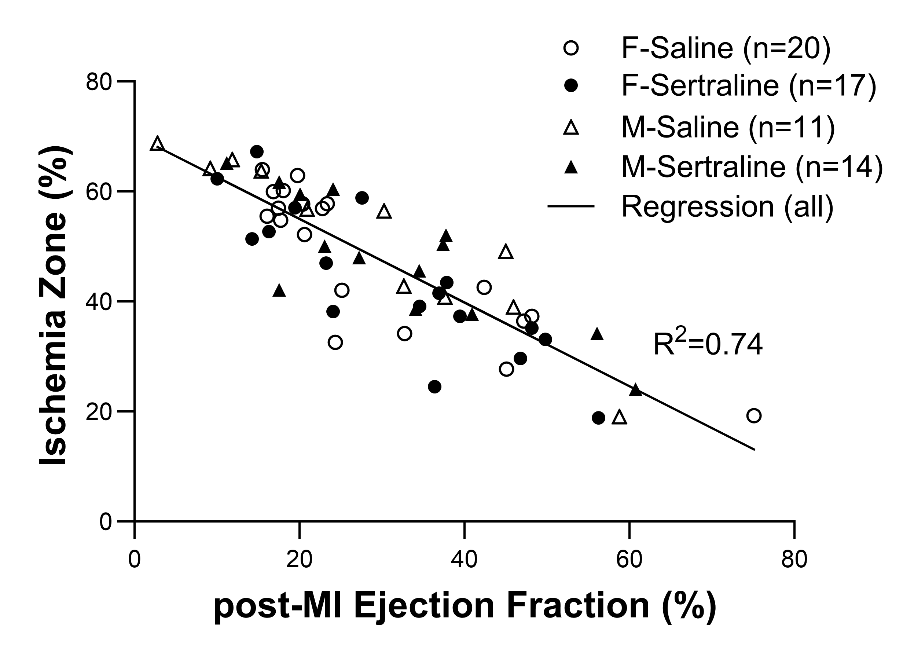


Supplementary Figure 1. (A) The MI incidences were determined from echocardiograms with the criteria of >30% for MI incidence. (B) Ischemic zone fraction (IZFx) is proportional to post-MI EF with correlation coefficient R^2^ = 0.74. No significant differences were observed between sertraline and saline groups, and between female and male groups (p>0.05). Mouse numbers used in each group were given in the figure. Statistical analysis was obtained using Fisher’s exact test for MI incidence and unpaired & two-tails t-test for Ischemic zone fraction.

Supplementary Figure 2.

1.
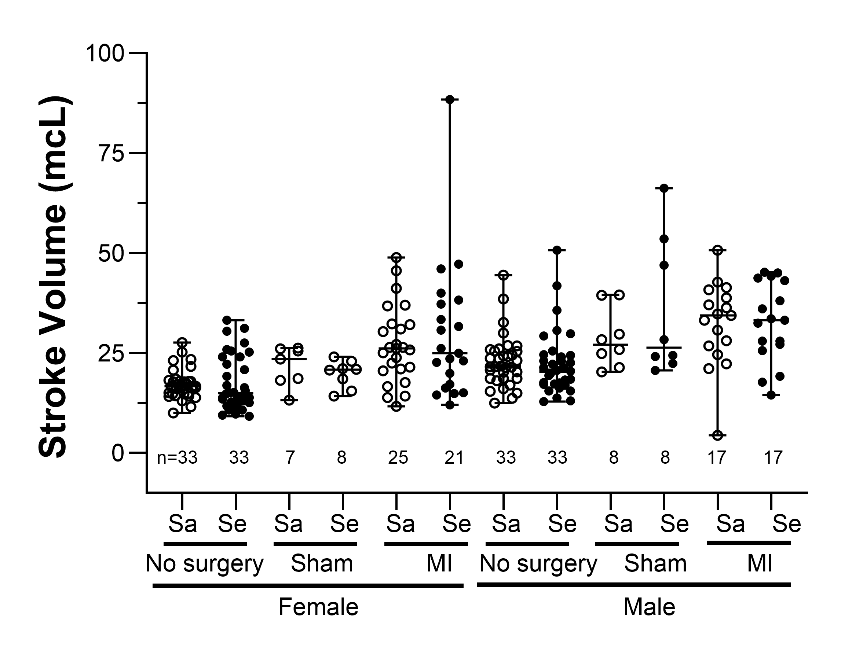

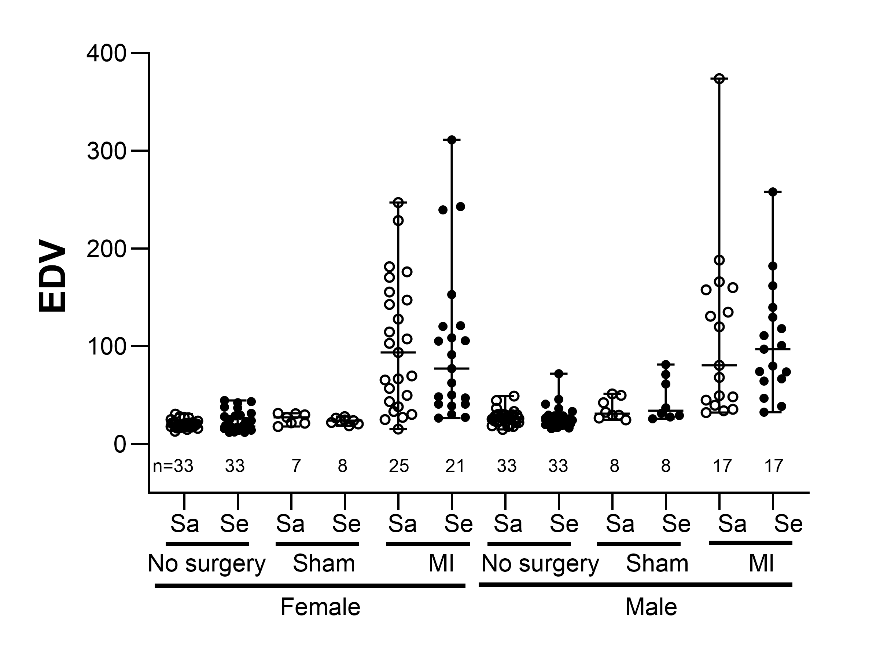
 B.


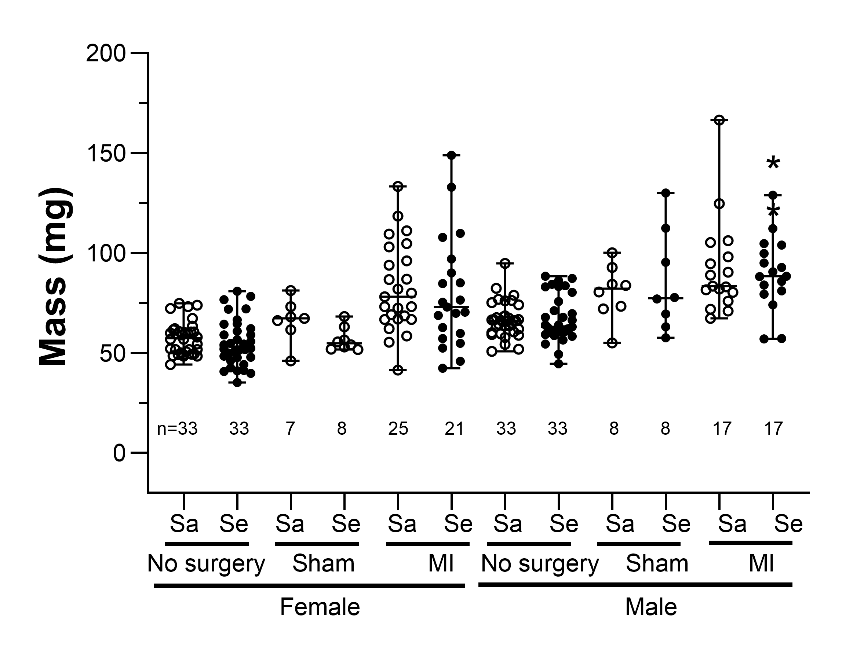

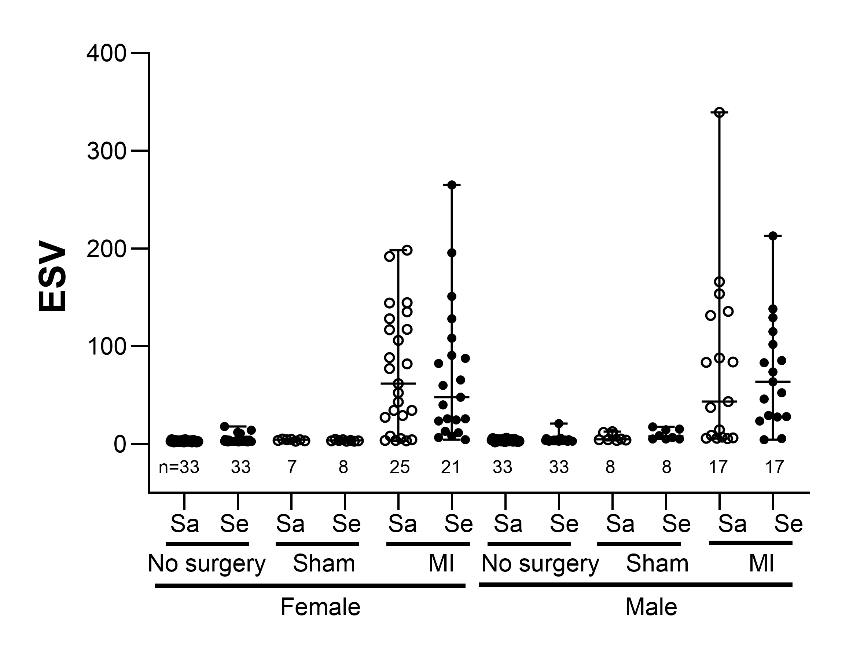
 C. D.

Supplementary Figure 2. (continued)


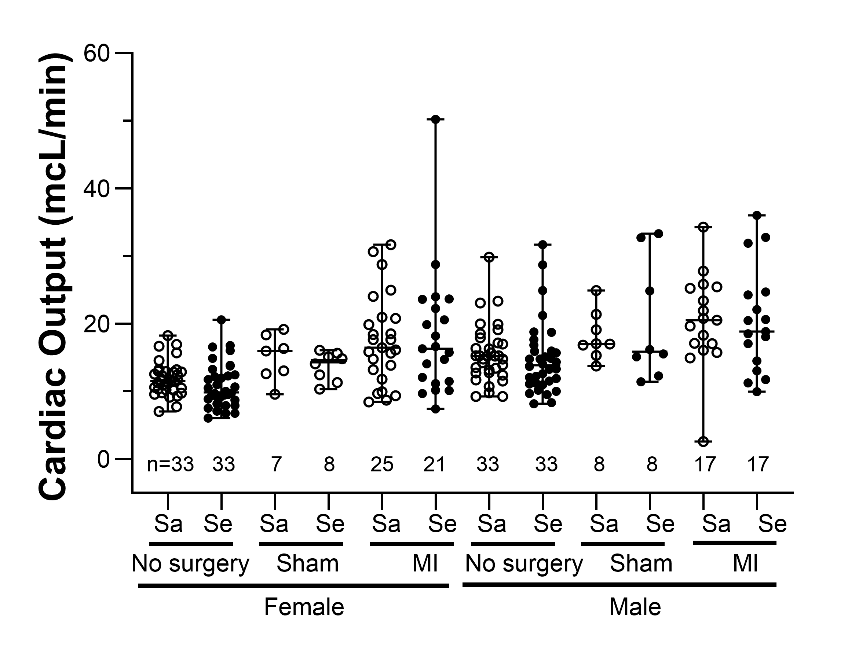

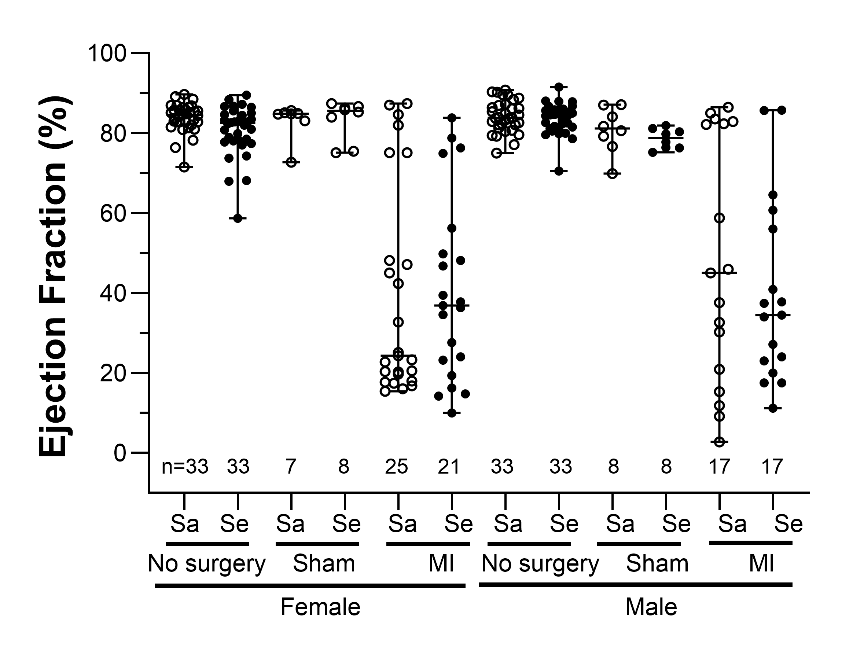
 E. F.


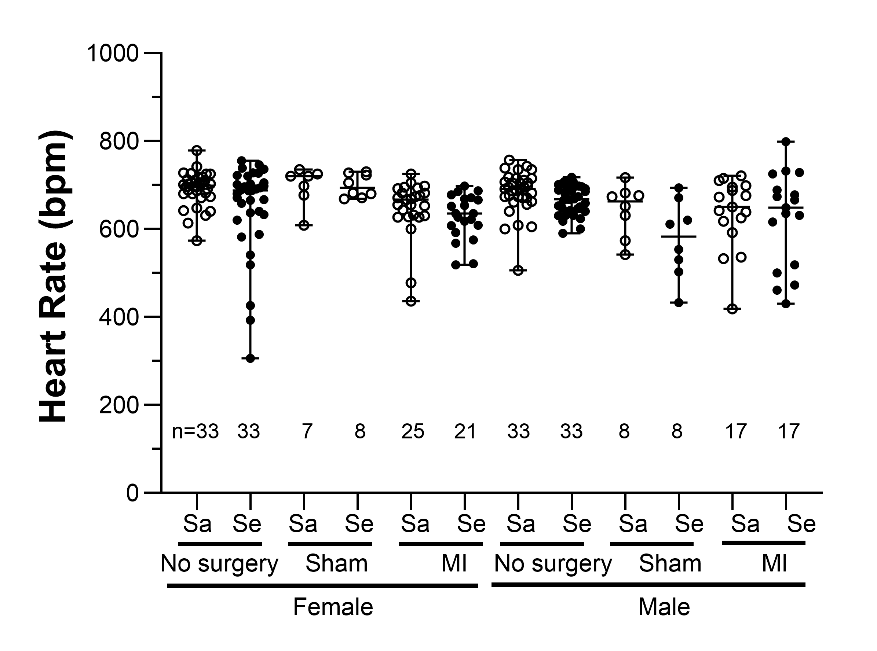

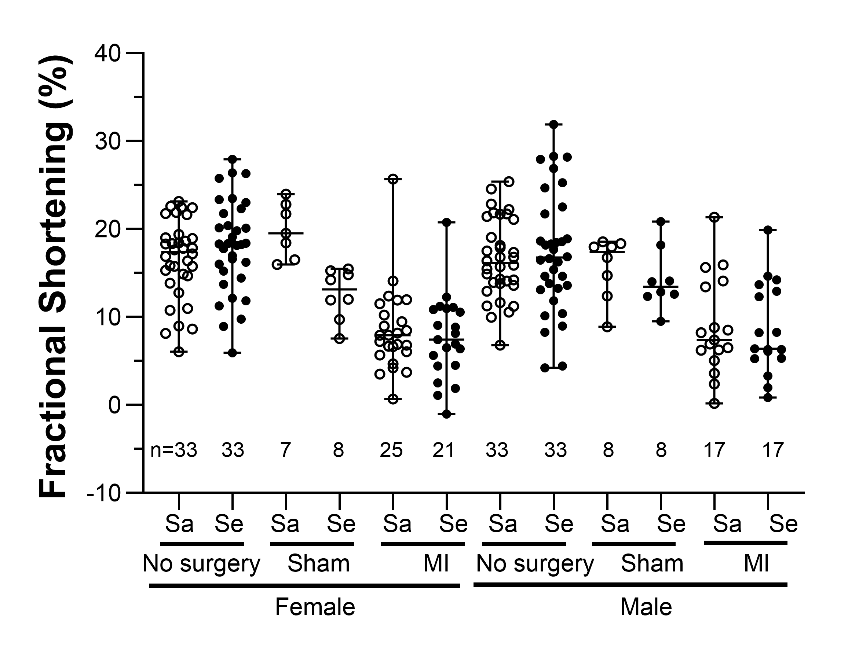
G. H.

Supplementary Figure 2. MI significantly increased LV remodeling in mouse hearts when compared to mouse hearts of no surgery group whereas no significant effects of sertraline (Se) vs saline (Sa) were observed. (A) LV end-diastolic volume (EDV), (B) LV end-systolic volume (ESV), and (C) LV mass (Mass). The functional parameters were calculated including (D) Stroke Volume (SV), (E) Cardiac Output (CO), (F) Ejection Fraction (EF) and (G) Fractional Shortening (FS). (H) Heart Rate (HR). All measured data points were shown, with the mouse number indicated in the figure. Statistical analyses were reported in the main text and Table 1.

Supplementary Figure 3.

A B


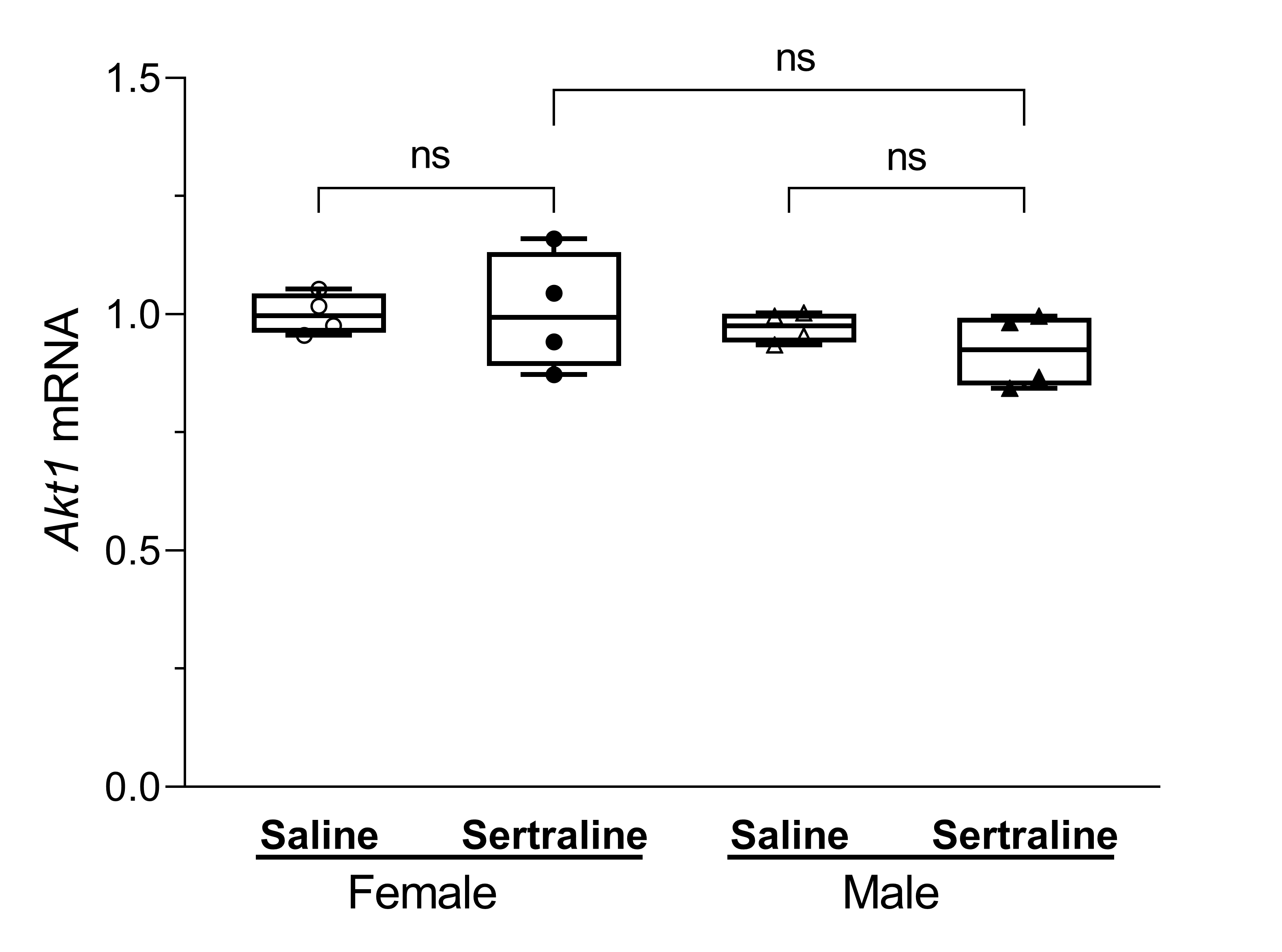

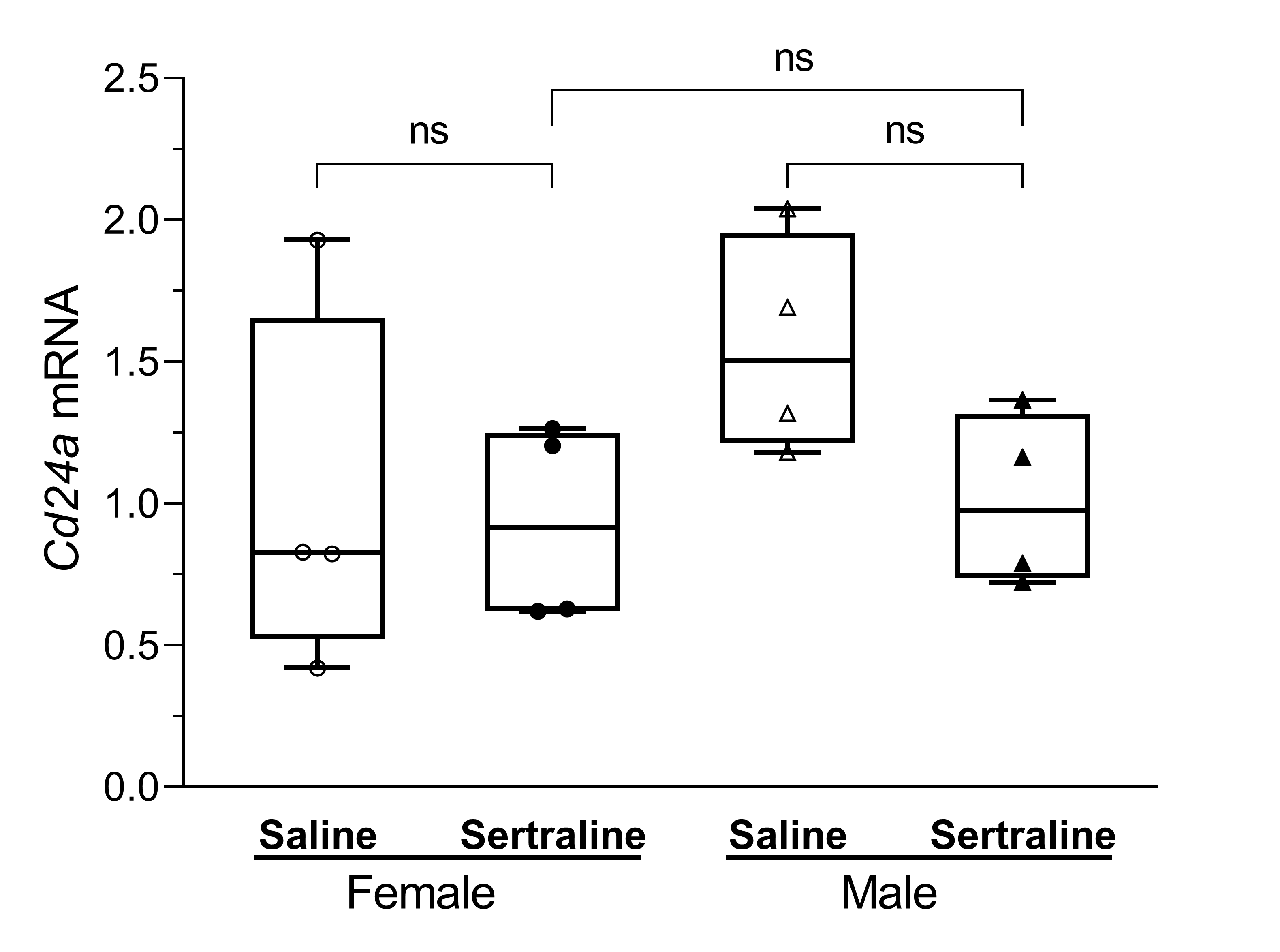


C D


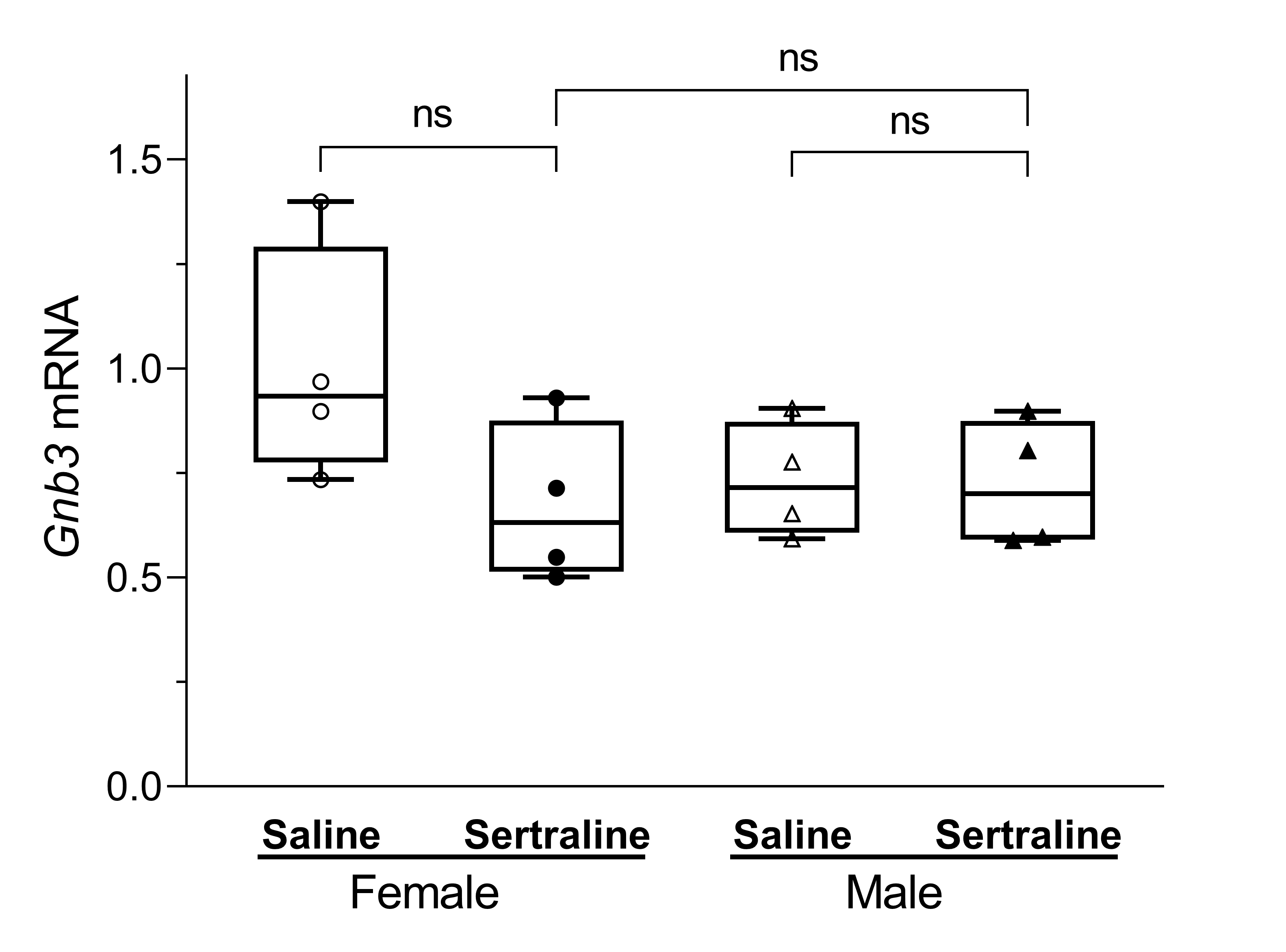

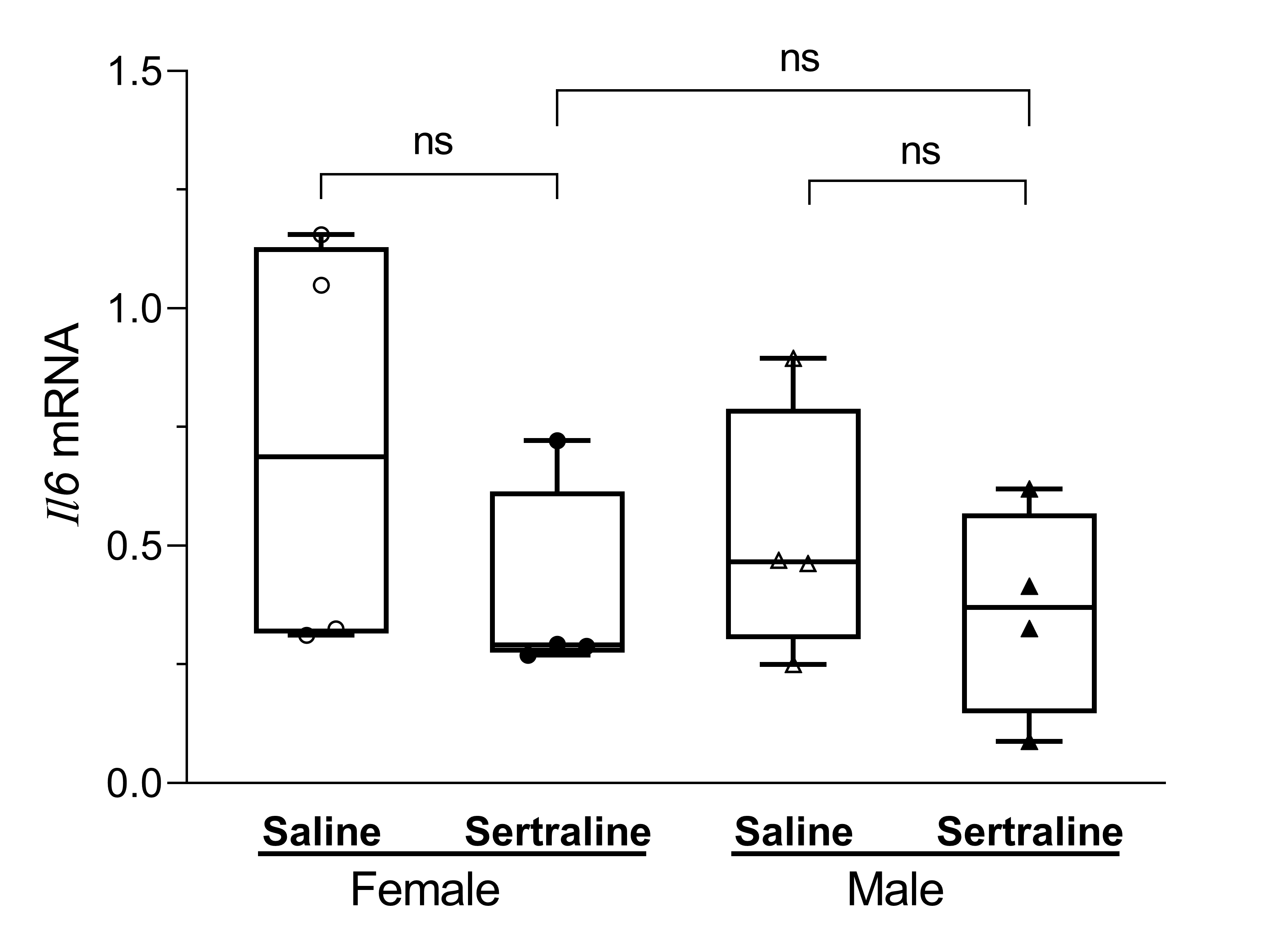


E F


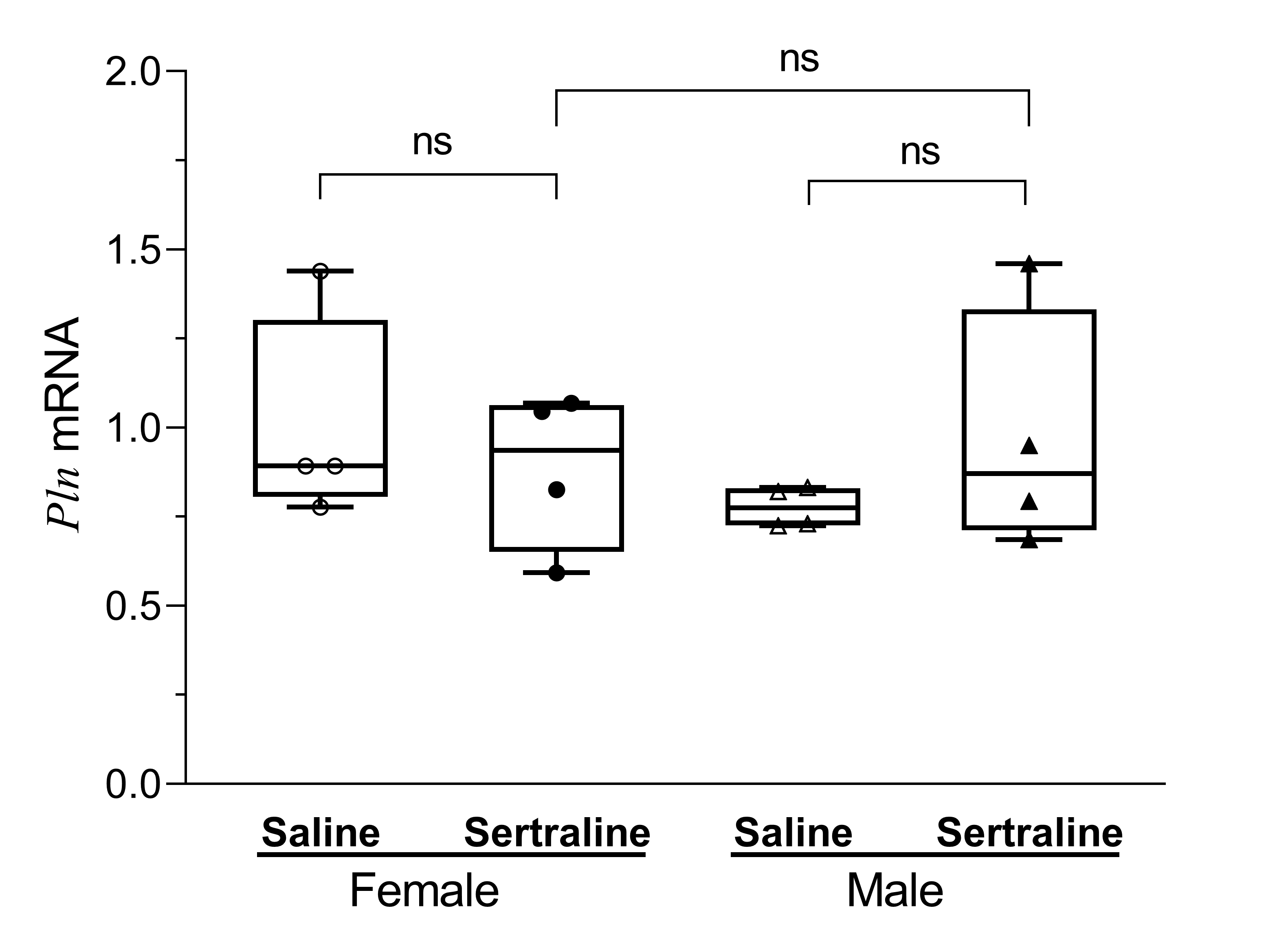

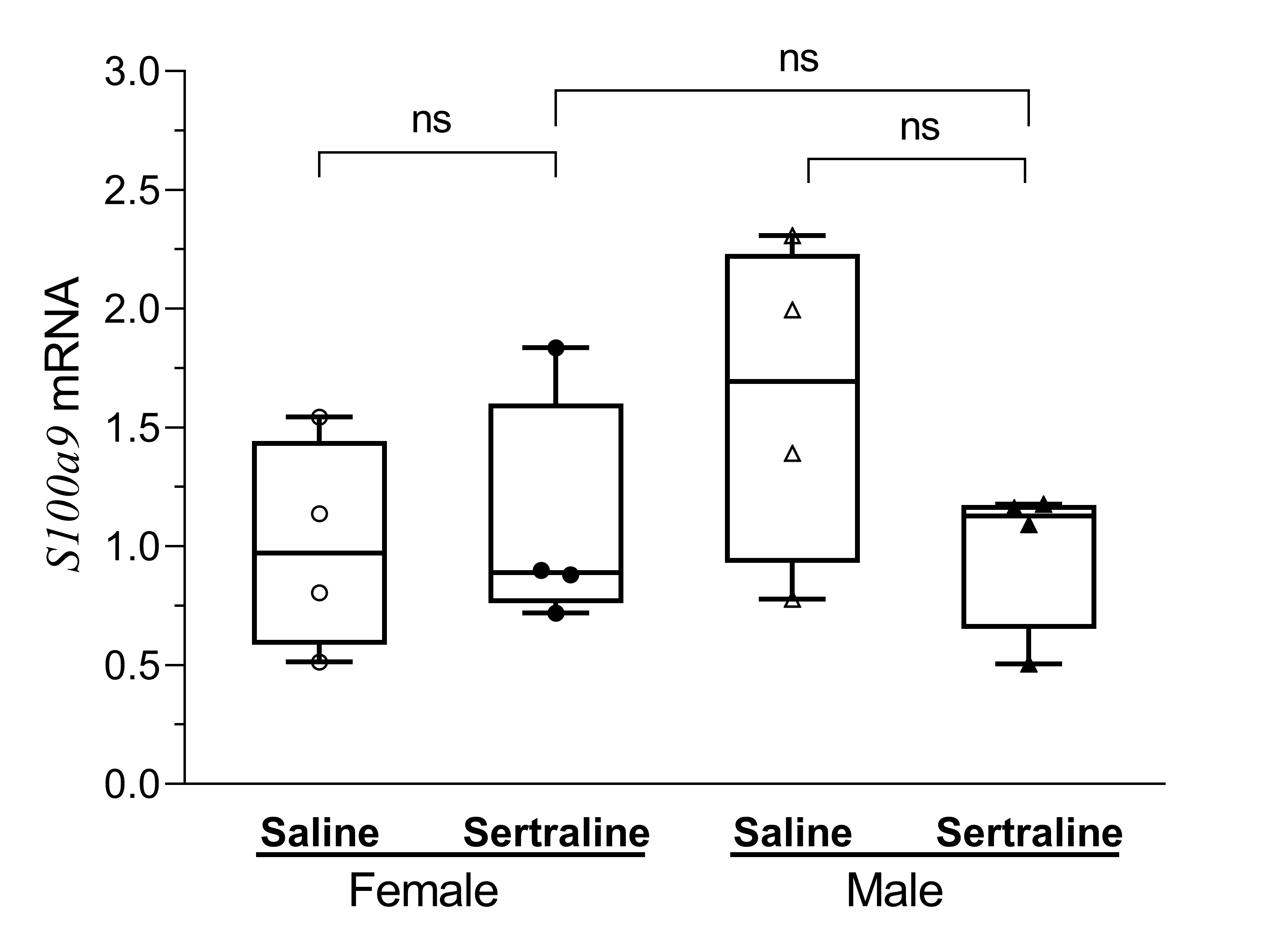


G H


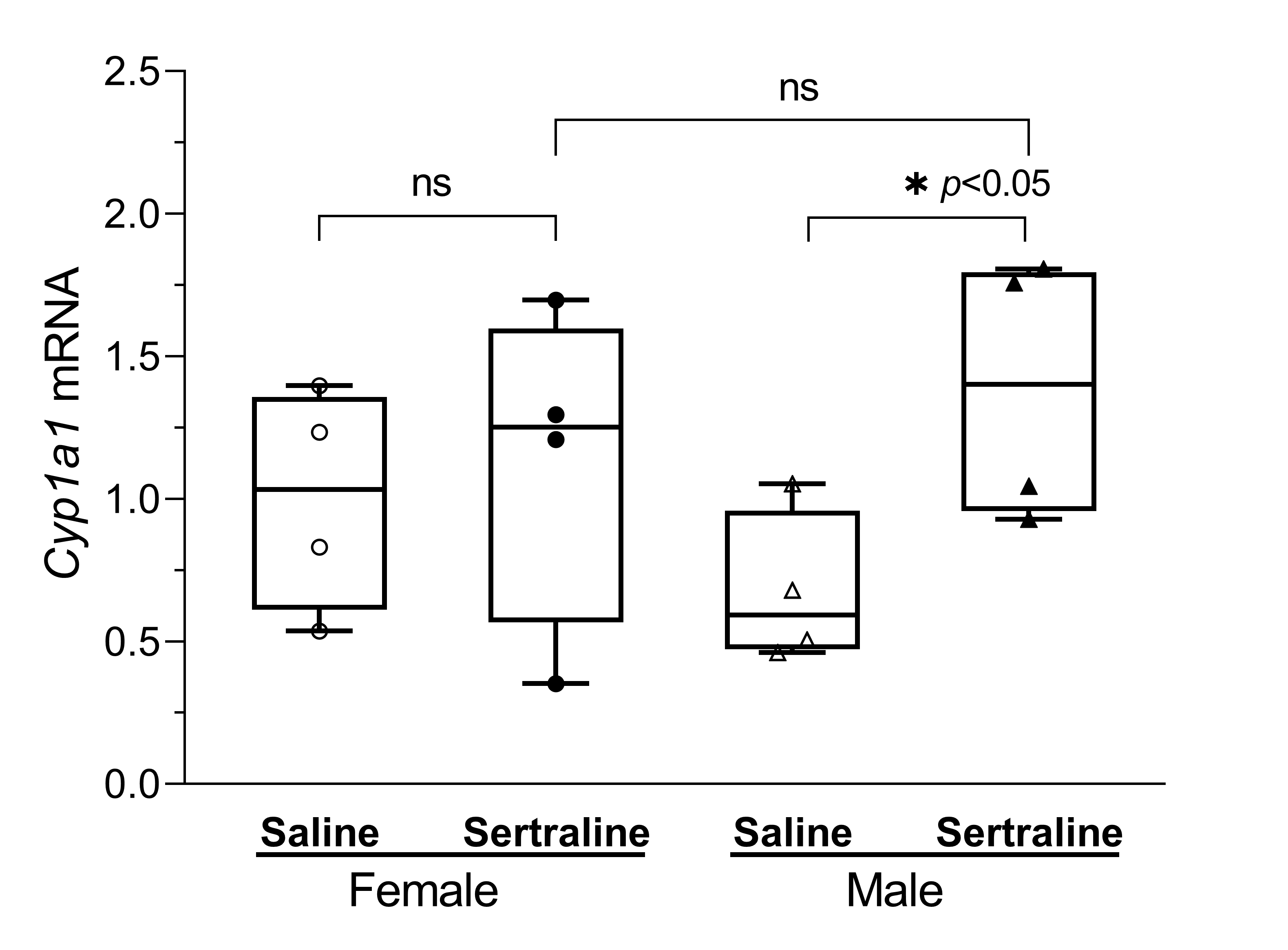

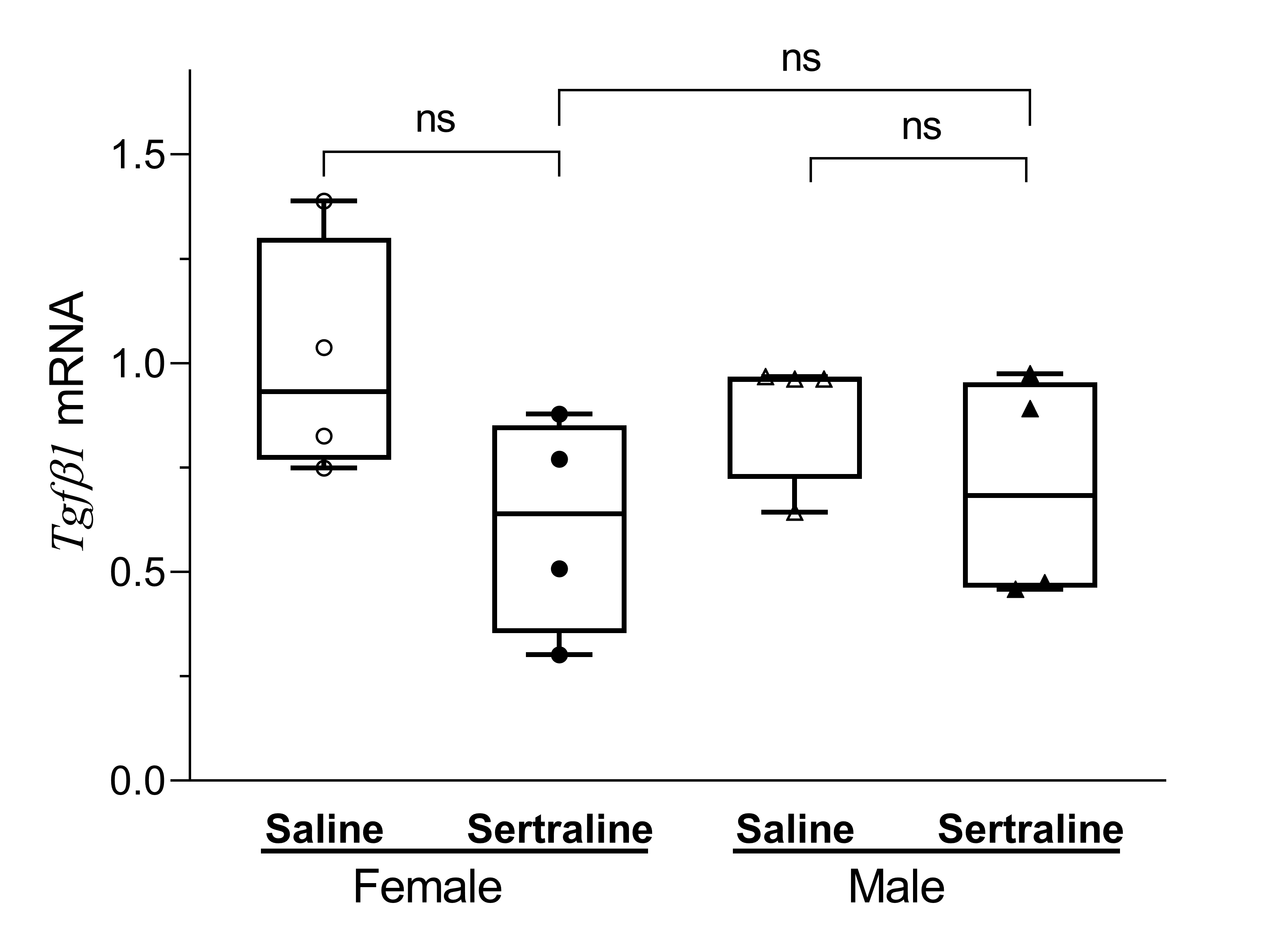


I J


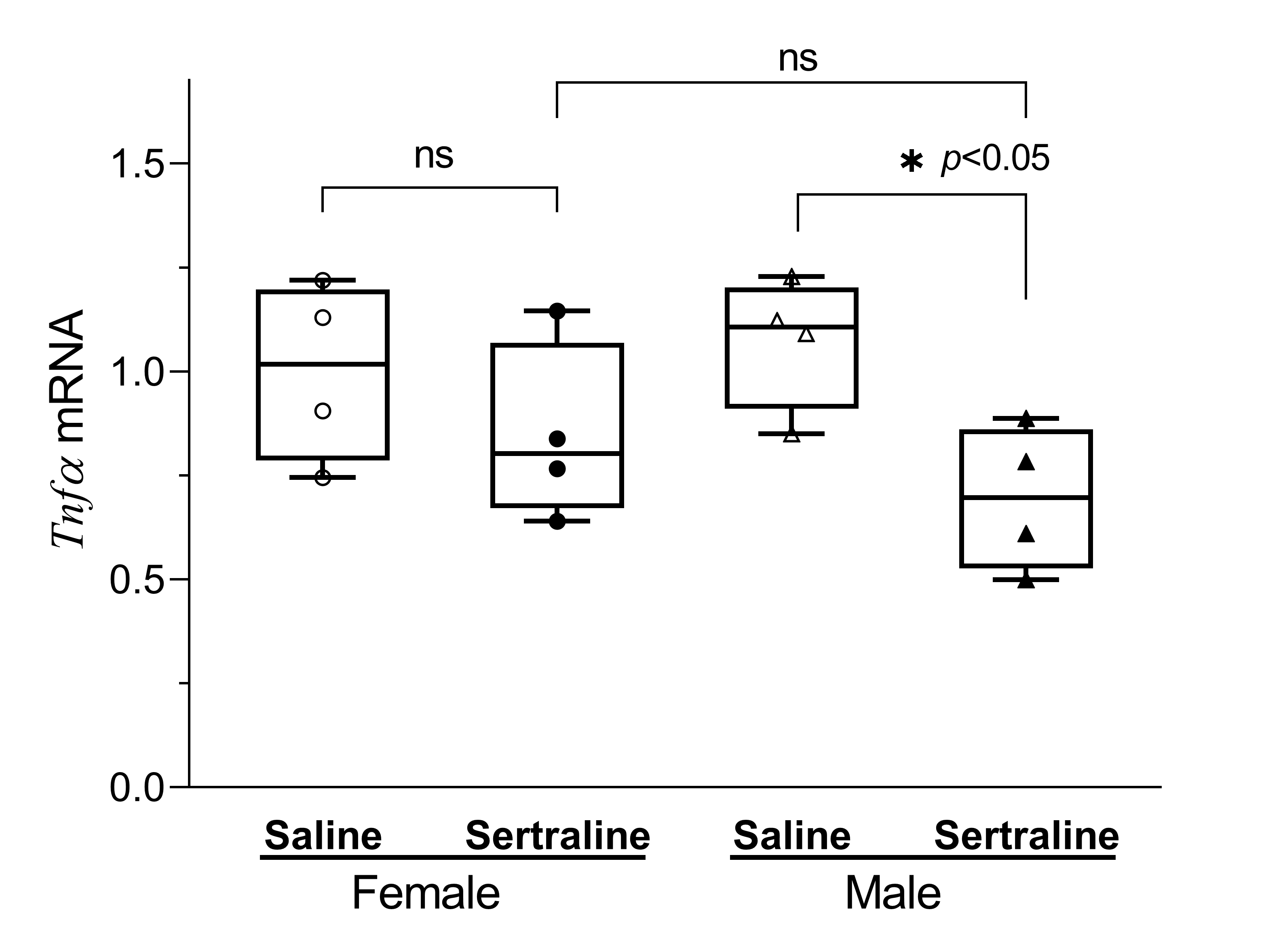

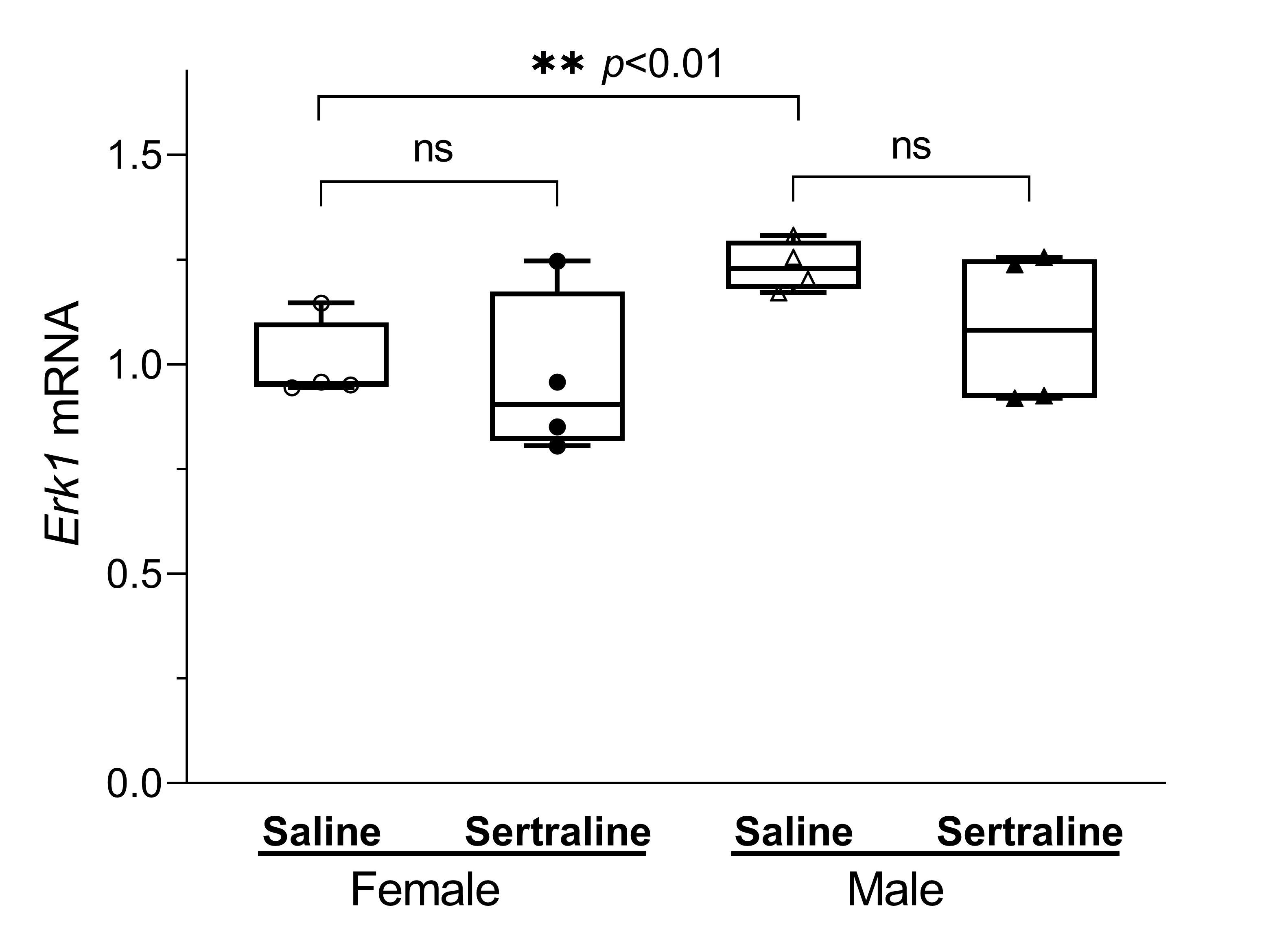


Supplementary Figure 3. mRNA expression other genes in cardiac tissues of female and male mice post-MI (n=4), which play critical roles in various aspects of cardiac physiology. (A) *Akt1*, (B) *Cd24a*, (C) *Gnb3*, (D) *IL6*, (E) *Pln*, (F) *S100a9*, (G) *Tgfβ1,* (H) *Cyp1a1*, (I) *Ekr1*, and (J) *Tnfα*. The effects of sertraline vs saline in each group were analyzed by unpaired & two-tails t-test, and the p-values were given in graphs.


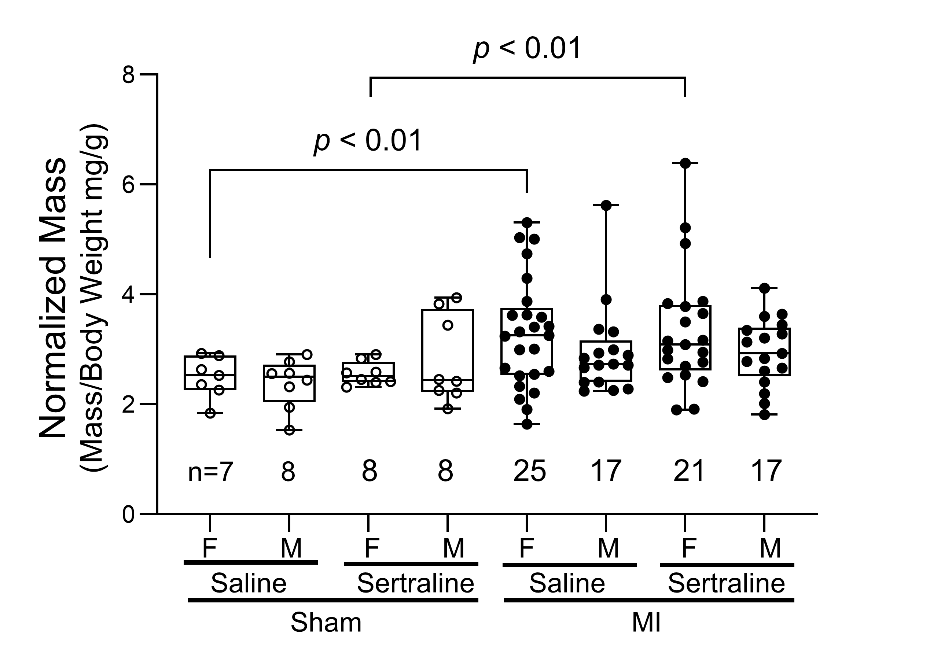


Supplementary Figure 4. Individual mouse LV mass normalized to body weight. Mice were grouped by Sham or MI, and within each surgical group by Saline or Sertraline treatment, separated by sex. Sample sizes are indicated in the plot. Normality was assessed using the Shapiro-Wilk test. Comparisons were performed with unpaired t-tests with Welch’s correction for normally distributed data, or Mann-Whitney tests for non-normal data. Mouse numbers used in each group were given in the figure. No statistically significant differences were observed between sertraline- and saline-treated mice within any group.
